# Supplementary material for: An ancient yet flexible cis-regulatory architecture allows localized Hedgehog tuning by patched/Ptch1
Source: eLife. 2016 May 5;5:e13550. doi: 10.7554/eLife.13550 (PMC4887206; doi:10.7554/eLife.13550)
Supplement: Supplementary file 1. — Sequences were taken from the dm3 build of the UCSC genome browser. Optimal Ci binding sites are highlighted in red, low affinity Ci binding sites are highlighted in gray and further annotated with a numerical ranking corresponding to the affinity predictions previously defined (Hallikas et al. 2006). Blue text indicates how each Ci binding site was mutated, for example in ptc-prox enhancer DB, the optimal Ci-1 binding site of GACCACCCA was mutated to GACaCaaCA. DOI: http://dx.doi.org/10.7554/eLife.13550.021 [file elife-13550-supp1.docx]

**Supplementary File 1**

***ptc*^prox^ enhancer (DB) chr2R:4,536,264-4,537,090 827 bp**

ATGCATGCGCAGCCTGCCACgcacgcgcttcccccaaacaaatacacacacacacactgagacgaaagctccattgggcagcgctgccgacgctgaaggccgacatcggcagagctgaacgtttgggtaggg**gaccaccca**catcgcttggcggtttcagtttaatgaaggcagaaacaaatttatttt

**---A-AA-- Ci-1^ko^**

**tgggtggtc**cacactgcagcgaaaataaactacagtggcaacaacaaaccagcagccaaggcac

**--tt-t--- Ci-1^ko^**

tt**tgggtggtc**catgcaaaaaaaaaacaaattacggcatgcgaataacaatagaaattagcgct

**--tt-t--- Ci-1^ko^**

ctcgtggcggagctatttgggtatattagagctacatattttatttgtttataaaaagtataaatgtaaacaatgagttccaagcattaagtccgtatgctcaacaattacattatcattattattatcacttaaatatttacaaaggatatttaaacagtaatagatatatattttatttcttaatttctgttaacatatgtatttacattggtagttattctttattttgcaacaagcattcataaattttatataacaaacttggtattttctcggaaaaactcctgaatcacccctcggtattttgtgcgttgagctatcgttaaagcagccctcgcagagagcgttctcaaaccaaaatggccgcacacgaaacaagagagcgagtgagagtagggagagcgtctgtgttgtgtgttgagtgtc**gcccacgca**cacaggcgca

**ci-63**

aaacagtgcacacagacgcccgctgggcaagagagagtgagagaGAGAAACAGCGGCGCGCG

**VT chr2R:4,522,304-4,523,363 1060 bp**

CCGCATACCCTATGATATGACTCGACAGACCCCGAAGACTGAGataataCaCtttCCGCATACCCTATGATATGACTCtgcttaaaaatgtaatggtactcaatcaaaatgtaaccattggtttttcttttaaatatcagctatagaaatgtcatatatccttaattaaaacaatattccaataatgtcttgctgtatgatatattttcctaatgtaataaatatagacttattattatgcaaattttcgctgcgtgttgctgtgtatgtgtgattggtgaatggcatgt**tgccaccca**agttgccagt**tggctggcc**aa

**ci-93^ko^ ---a-aa-- ci-141^ko^ --tt-t---**

**ci-1 ga------- ci-1 ---g---t-**

gacttaatagccgctttcattggccaaaagcctgataaaa**gaccgcaca**cgaagctcttcttcg

**ci-59^ko^ ---A-A---**

gagccaaggcagtcgtggc**tgtgtggtg**gtttcgtgggtttgatggttgctggc**ttggtggtt**t

**ci-196^ko^ ---T-T--- ci-203^ko^ --T--T---**

ggcggttggcctttcgacgtctcatcggcaacattgcgttcgccttgatgtttatcggcaatatttagctgcaacatgttttgccaaggcatcaaacggtagctcttcttaattacaacaaccaaaacaacagccagccagctggccattaagataaatcggaaacggaaatcgctaatgaaaaatttccatatttatggtaacgacaaagagccgcttcgctgcaaaggcaaaccgaaaaccaaaagcaatagcaaaaacaataacaaaagcaacaaaaacagcaacggcaaccactaaacaactaaatttcgttgtcgactgattggggggatgtggatgt**ggccaccaa**cgacgaaccaaccact**tggggggtc**cagcctc

**ci-47^ko^ ---A-AA-- ci-5^ko^ --TT-T---**

gcgtgatttgttctctttttttttttttttcattttcattttatttttttctggcccgccgttttgcttttgggccccgtctcgtgtcttta**ttggtggta**cgggctgcaattaattccaacgaaatt

**ci-195^ko^ --TT-T---**

taattagaattaaaatgtatatttcattgactgggcgcgataaggcagcgcaaatgtcaatcaaacaaatgggtcccgagaatattaggcgattcgaatgctggcgcgaaagattaatcataaCTCAGTCTTCGGGGTCTGTC

**LK chr2R:4,530,049-4,531,613 1565 bp**

CTACTTGGTTTGATAAATACGGTGTGTGTGAACCCAACTAATTGCTACTTGGTTTGATAAATgggcgcacgcctagagcgggctaatcggaaacttccggcacattggagctcataaataacgaccattgtgcacctagaaaaattatttaaatgtgatctaagttctaagtttctaagcaaaatgggttatatgtaaatgatgataaatatgcaactgatctgagaaatttggataaagtttgatggactttatttggagagggaagagaaaagttcacatttactttaaaaaataattgtatcacaaataaaagagcgagaaaagagctgatattgaatattattgttaaattgaatatatacttttcctttaaaaaagcattgcatactttgcaacagcattaaaagatacttcaatctcctgtgtgctgaaattgagaatgtatacatattgaacgtattaattaaaacaagaagctggaattatgatttaaatttcttatcgtaacttccagtttataatgggtcatagaaagtgcctatttttc**tgggtgcac**tgctggccaaaacaagt

**--TT-T--- ci-100^ko^**

ggacga**ggccaccaa**at**gacggccca**cgcctgaccccgactgcgatgacccagggacactgtcg

**---A-AA-- ---AA-A-- Ci-47^ko^,Ci-183^ko^**

ggcttatcatgacccgatattggacgacgtgtgcgggccgcccctgcccccgcccccctcagctggatcaattaaaacgcaggcgagggcagcgggcggcgggctaacaactcaatacaatgtaaacaaatgatatacggtcgcagggccaccacagagtcagggtccatttccattcccatttccatgcccatttccagtttgagctcgattttcattttccggagcgattcagcgattgaaacgtctctaccttttccacaaaagcgacagccgcacagctggactcggccgactggctcagcagtcgctgcaaatcaattcgaaagagattttcatttgatccgagctcgagtttgagtcgtaaaaagcgggcccgtggtcaggctaacattcgactggtgatggtggtggcctggtggtcccggccgatgtgcagcgatcgtatctggccagatatgtcggcccaaacaagttgagttgtcaacgcagctccgcaggccacgtgaagtgtcgttgagttatgactgtcccgggttttaccctgaatccaaatcctaatccgaagctgaagccgcagctagctcaatgttgggtctttacgactctgccggccatgtggctaattgatcgtattaagctgtgggaaaagtagtttgggtacttgctgattatacattgctgattgcacg**tgggtgatc**ctc

**ci-16**

ggccag**gagcaccga**ccgtccaggtgtctttttcgattggatttttgaaagtgcacaatcgagt

**---A--A---A-AA-- ci-174^ko^/184^ko^ (overlapping)**

cttgaccacaacatagctttcttctcgttgccagtggggtgttgagctaagcaaatgaaaagaatgttataaacaaatgactgtctaagcttacacatttgtaaggctttataatcgtgctc **tgccaccca**ctcacacttggagccacacaatcttcggccgcaagttcaacattCAATTAGTTGG

**---a-aa-- ci-93^ko^**

GTTCACACACACCGT

**1EH chr2R:4,542,479-4,545,408 2930 bp**

GAAGTGCTTAACAAGTTAACtgtaatttgacatgctgggcgtgttgaaagcaaaacacgataaggtgtgcacgcccagaaaatgtccacttctgggaaatgtcatttaaatacaatatgacctgaaaaattcgtagccattccatacaaagaattgttagtactgaagattatgccaataccaatgggtatatagagtcgtagttccaaaagactttacatgaactttaatcggattggagttcattcataaaaacgcatcactgccgctttacataatcctttgacccattatcaggggtttgaacttattttccccttcgaatgaacccaatggcccacataactcccacacagtgatcccacaattgacagcttcatctgctgcggccccacacactcaacgcaccaacgtgtccaacttgacttgatctgccatctgcagctggcgcggggaactggaactcaaaaaaaaaagaaaagaaaaacacaaaatacaaaatacaaaatgtgagggaaaaaatgaaaataaaccgaggaaaaaacacgaaatgctgttgtacaggcggccacagcttgggcttcattagggacgggacggcaacgacttttgcccgagagacgagccgcacataaaactgttattgtcttcatcagtctgaggtctcggcgaagcaagacataacaacggcccgacagagagagagagagaagaaaaatcgggagaattatgaagacattaactc**gaccacaca**gcacgctgccgtac

**---A-A--- ci-9^ko^**

ccgtacctatatacctatacccaacccataaccatacacacacacacacacacacacgcatcaacacacacacacacacgaacagacaggcccaaaaactcgaaaccccagcagagagaagcgggggcttacttacgttacgatcaagacctagagccgagccagaaaaaggtatactgcagagacagagaggagggcacagtgacagagagcgaataccggaaagaaacattcaagcaataatcacggaattcactgaaaaagcagttaacggacagaggtccacggacggactggcggacggacaaaactccgctgaacggacgcgaactcgatacgatacgatacgatatgatggcaaaccatcggcagtggagaatgagtgcgtgtgtacctgtggcgggccaggtaggttgcgttttcgattttcagacgaaataataaagaaattaaaattgttacggcctctccagatcgcagggcaggtgaacttagcttcagttgaaatcgaagtgattacaaaacttttttgacagactgtccagagagagagggaagataatcgggtatccatgccggtggtcctcttctgttttgcacctggcgcttctggagttaactgtaaaccgtctgcctggccgctcacctgagccgcactgtgaccttcaaacctttttttaacgatttcgctttacgggcatgcaaatatgttttttttactcctttttttcgactcgctcgatgcaaaaagtaaagagcataaaatggtaataaatgcggcagacggtttgctagtcacgttttcggggcgccataaattggcggcagcaaggaatttacattcaacggtttcacattcgtttgggcctcatttttaattgcctcgaaatcaatttacttttgct**gtccaacca**gccgagtgcatg**tgtgtggtc**cttaatggcgcccaatagacggaaa

**---a--A-- ci-180^ko^**  **---T-T--- ci-9^ko^**

aattagtacggagcgctaaacgtaataaagaattccatatccctgctaaaaataaacaataaatag**gcccatcca**tgacgcactatatccatttccaccagctattttgtttgacagctgggtgaatt

**---A-AA-- ci-140^ko^**

gggatgtgaaactagaaaaaaaacacacttccattcaaatgtcgggcgggcgagatgggaaaaaaggggaagggtaaccaccatgagatacacgccagatcggatgtggcaatgcagaagatttcgtattcaaaaacgttatcaagtggcatataacgtttatggtagctacaagttttatggcacacgcttacgtgagaaacatgggaaagctgcgacactcgcacacacataacacacaaagtccaccggatcagagattccatatatagagcagtcatcagcagtaatcccttgagtcggaatcgaaagatatgaaaagattcacgcgctcgcacaatccataaatttggaccgcaatggaatcgcctgtccatatacacaatgccatagatgctccctattcatattcgattcgtc**tgggtggtc**tgccatatagagtttcatt

**--t-tt--- ci-1^ko^**

ttatgataaaaatgctgttttttgtggttttgcattcgatttttattgcggcataac

**tgggtgcgc**tcatatttaatggcccaacgcaccatatacgagccgagagactcaagtgcttttc

**--tt-t--- ci-83^ko^**

**------gt- ci-1**

ggatttccaaatggcatggaaaagttttctatatacatatatatatatatattttttttttcaaatttagagtggttcccgaagcggtccgtgtaattaagtagtcaggcgctg**tggggggcc**ggaga

**ci-23^ko^** **--TT-T---**

**ci-1** **----T--T-**

aaattcgatttttgccttttgccgcgccagttgatgtgtcggatcttttgattgattgatgcgtgatgcgaagatttcgagattgtttacagaagagaacagaaaacttcaaacatacagaattttaagtaaagtagctcacaattttcttttctcttcatattctagtagaatcctttatgatttcatagttaatcgtatagaagtatgttaatctgttaatatttaaaatcgagctagagagctgaatttttctccgtgtacttgtgatacatttctacccatcatctccggtctacctgcacctgctgttcccaactgttttcgttcttttcggccaccggtctgcccctagctCGATCTCATTGGTTGTCGTG

**HF chr2R:4,533,138-4,535,559 2422 bp**

TCCCACTTCATAACCCTCtgaaatctggacaagacacttcgattccagctaaaataaacctatggaggaaatgtcgccatgcgtgggttcgctcagtgatagccgaaaatccatccatctgtaccgcaaaaaatctattgaaaacaaaacccaaataacctaggattgtacactgtaaaaaaacgaacattttctattgaaaaagtttttgtttctataaatacaactgatgaaaggttttaaaaatgagctaacccaatttttcttatgccaaaatagtacaatacaaatcgtctccgattgattttttgaaatcgaagtatcccgaatttgttcagctttttgaccataatttttgccaagtgcactgcgagaagcagagctgaaagcatggcag**gaccacaca**agaagctgcaaaagccacacacaccgtcgaggggacgggggc

**---A-A--- ci-9^ko^**

agactgctcagttccccagttcctcagttggtgatgtcggttgccggtcgccggtcgccggttgccgttggacgtggcggttgattacatgtacaaacgaaccgagccgatccgatccgatccgagcccagttccaggcccagtccgatctggtcccccagatcgcagagccaaccgcaaaagccgacgccaagataacgagttaacccaaacccagaccgcaggcaccggggaatcgggatgacgggccatcgggacaaggggagtcgcccg**aaccacaca**cgccatgtcgctggggattcggaatcgcggcctacgta

**---A-A--- ci-196^ko^**

ttaccggtggcaaatagtaattattacattaaatgcaccgcaataatgcagccgcgacgtcaacatttagccataaaaattgcttgcactccgctgttggaaatgcagttaagcttaaagtggcggtgcaagaggtacaccgaaaataattacaaataccaaatgttatgatattatgatagattaaacctttaaattgatatatttatggcattactatacatcaagtagcgaatccgaatagaaatagaattgacttcactgaatcacgtgaaagatctctttaaatttttttttttatgtaaaccttgtgaatgggttgtggcggtggaaaagggggatctagagaaaacaaccagccgggtaatggtaatggcgctatgggccaatgacaaatgcaatagcgttggtttggtaatcggggagcgggacgagaggattgtgtatcagttggggtctagggaacgctgcactataaattaagacattatacgattaaaaacaaacagcctgtcgttaaagtcgccaaaggaggaaaagagagcggagagcggagcgagcagatcagggttagaatccagcttaagcaacgggttgatgggcgagaccaaccgattggaatcattcacattttatagtaatggtaagggtatgttgcttaatgttgagaatttagtaaaaattttaaatttatttaaatgacttttgccacaactctactcccttttccactaagtctgcctcccgaaaaagctaaaaaaaaatagtttttgaatgcggggttcttaggagctttgtagagctcttaacgcttataacatgaaaaatatttatatttggtttatcgctttaaacgatcctgaattgagaaatatagattgaaacagaattcattaccatttaagaatatcattatttatgggggagtaatgcgcctccgagtaggcaatgcttttcttgacattgttactaagaattgtgaatgatatttgggcgtggatcaacgccgattaaaagctgcttttgcttccaggcggccagagaagagatccaaacttcaactccagccataaaagcaacaacatttccgtctcccccttgtagctccccttcctccggctcttccactctccacgaaacggcaaatgaagctctcaaagcgaactgtgcttcgctggtggtccattggcagctgccgccacacaggcgctgcttttgtgtgtgtgtgaatatcaatcttgctctccctctctttttatctctcttggggaattggagctgcatgcgaattgagcgacagcaaaacgaactgcaagtcattgagaggagagcaaaaactcgagagcaagccaaagatggcgcaatctggggagagcgaaataaagctaaaatatgcatgttggagaaaaaatgccgcccatgtcgccaaaatgcgccacacgcagagtgagcgggcggaggtgggagtaatggaaagggcgatgagggaacgattagcttgaagagagagaacaacaaatgaatgtgctgcaacgttagttcaggtgagcgagttagagagagagttgttgttttttgattgtaatagctcgc

**ttggtggtgggtc**cacattcacatctccctctcccactctTTCTCCCCGAAAGAGAGAGCG

**---T--T------ ci-202^ko^/157^ko^ (OVERLAPPING)**

**YU chr2R:4,520,384-4,522,692 2309 bp**

GCCCTGTCGTCTTTGTCTTCtttattcccagcgctcc**tgggcgatc**ga

**ci-106^ko^** **--TT-T---**

cg**tgtgtggcc**ctccacatcccatttgcgatcggagcggattgtggggcattattggaccctgt

**---T-T--- ci-44^ko^**

ctttgtgccgcgatttaaacatacatattcggttttctaggaatcgggcaatcctagtgcgccaaacgttcggaaagcttggctttattattatcgttccgaggaaagcgaaattaggtttttatttcgggtagggcctgggaatgcggatggaggttaaggtttggagaccaagcacacacccagaagaaaaaaacttttttaacattttcaattttattttgcttattttatctttgctcgcagccataacgtcgagatcttccagggtgctgtatccgtggtgaagggttcggggggca**gagctccca**ctattaata

**---A-AA-- ci-88^ko^**

ctacaattttggaaaaggtttattttattattcttgattatttccattggtatttaaaaacaatattctttctggtactcacactagctagatagtcgagaaacttatagctcttactgtcgttttttattgataaaaaaaattcttgaactttttttcgccgacagcaatgagactgctccaaaaatggatatccctaattcttttcgaaaagtatgtatatattgacttttttcagtttcattgatcgaattcagccaaagactgacgttttacccattcacgatctcatcttcggtcttagacagcaaggaatgggctgcggttagttaacttaggggtaatcatcagccaaatgataaaggctagatcctctactatcagatacctattactcaaataatttgttttcatacaataaagatagtcttttatcctgtatgtaaatcatttatgatttatatgattaaaaccaattttcgtcccgttaataaaaaaaaaaattatttataaaatctttaaaaatataacagtttataactattacagcaccaggtcaacttcttcgatcccatctcggattttttcaagccgttcttctctgtaattggcccaaagaacgtagaattgcccagcggcagcaataaaattatgacaagcaacggctgtaacatcaaatgttggcaacaattattgcgttgttgacagcaagatagatagcaatcatttgcatcgccatcgctggaaaaaccg**aaccaccaa**cagcaa

**ci-199^ko^** **---A-AA--**

ttgtttgcggctgttgggcgtgtaatttacagcgccaagatcaaaaaacaagatgcatgttgcaagttgcagattgcaggcgcaacaaaaggggagcagcacacgagcaacatgttacttttgacaatataacgcaacggcgtggcgacaaccaactgggagcatctcggatcggccgtatatatatgtacatatctgtatatatgcacatatgtatgtacatacatatgtatcccccagacatcggaggcattcggatttcacggtggtcccggcatcttcatcccaggcttagtttctgctgtcaatctttggcaaaaggcaaatgcggctactgtttgccgcacacacaggcacagatacagatacagatacagatacaaatacagatacagatgcagtcccatcgtataattgcatatagcgccagtggaagttccggtcggttggcttttttttgcccgttgcacgcgctttcttttttgtgcggcgccgccgacagacggggctacactgagagaaataattgcattttgttagttttaggaatcttacgaattcgaaattgtaattctaattatcacttagatactacaagatcatctatatatcttttgagaatatcatatttgctttctgaaaaaaatgatttttaaatttcttcaaaatttgttttacttatttaactattctatatatatgatttgcacaaacttaaatataatacactttccgcataccctatgatatgactctgcttaaaaatgtaatggtactcaatcaaaatgtaaccattggtttttcttttaaatatcagctatagaaatgtcatatatccttaattaaaacaatattccaataatgtcttgctgtatgatatattttcctaatgtaataaatatagacttattattatgcaaattttcgctgcgtgttgctgtgtatgtgtgattggtgaatggcatgt**tgccaccca**agttgccagt**tggctggcc**aagacttaatagccgctttcattggccaa

**--TT-T--- ci-93^ko^**  **--TT-T--- ci-141^ko^**

aagcctgataaaa**gaccgcaca**cgaagctcttcttcggagccaaggcagtcgtgg

**---A-AA-- ci-59^ko^**

c**tgtgtggtg**GTTTCGTGGGTTTGATGGTT

**---T-T--- ci-196^ko^**

**ZY chr2R:4,519,864-4,520,686 823 bp**

CCGGATCGACCTAGGTAAGGgtaaggatcctggtccagaatatatatactgttcctgtgacttagcattgaaaaaatcttgtatatatagtattcccattaaaataccttaaaccctcttctaatataaatcccatcttccattgctcaaaattgatacttttttttctcactgtaccatctcgctcaccaacacacatgcagcaacgagaaagagagcaacgacgtgtctcttcg**tgggtggcc**cttaattcgac

**ci-3^ko^** **--TT-T---**

gtcatcttct**tgggtggtc**catattagccggcctgctctctccccccttttgatctcaagtgcg

**--TT-T--- ci-1^ko^**

agcgagtgcgatgcatgtggataggttccccattgatcattttctcgtttgatagtgcgtgacgtctctgggcagccgtctcgctcgcactaatttatgccgcccgaatggtggggcgcccattttttgatccttcttctgctgctcactcgcgctcatttattcgagcataaaattagcaattatttgtttttcttgccgccctgtcgtctttgtcttctttattcccagcgctcc**tgggcgatc**gacg

**ci-106^ko^** **--TT-T---**

**tgtgtggcc**ctccacatcccatttgcgatcggagcggattgtggggcattattggaccctgtct

**---T-T--- ci-44^ko^**

ttgtgccgcgatttaaacatacatattcggttttctaggaatcgggcaatcctagtgcgccaaacgttcggaaagcttggctttattattatcgttccgaggaaagcgaaattaggtttttatttcgggtagggcctgggaatgcggatggaggttaaggtttggagacCAAGCACACACCCAGAAGAA

**GB chr2R;4,534:284-4,537,090 2807 bp**

CTATGGGCCAATGACAAATGcaatagcgttggtttggtaatcggggagcgggacgagaggattgtgtatcagttggggtctagggaacgctgcactataaattaagacattatacgattaaaaacaaacagcctgtcgttaaagtcgccaaaggaggaaaagagagcggagagcggagcgagcagatcagggttagaatccagcttaagcaacgggttgatgggcgagaccaaccgattggaatcattcacattttatagtaatggtaagggtatgttgcttaatgttgagaatttagtaaaaattttaaatttatttaaatgacttttgccacaactctactcccttttccactaagtctgcctcccgaaaaagctaaaaaaaaatagtttttgaatgcggggttcttaggagctttgtagagctcttaacgcttataacatgaaaaatatttatatttggtttatcgctttaaacgatcctgaattgagaaatatagattgaaacagaattcattaccatttaagaatatcattatttatgggggagtaatgcgcctccgagtaggcaatgcttttcttgacattgttactaagaattgtgaatgatatttgggcgtggatcaacgccgattaaaagctgcttttgcttccaggcggccagagaagagatccaaacttcaactccagccataaaagcaacaacatttccgtctcccccttgtagctccccttcctccggctcttccactctccacgaaacggcaaatgaagctctcaaagcgaactgtgcttcgctggtggtccattggcagctgccgccacacaggcgctgcttttgtgtgtgtgtgaatatcaatcttgctctccctctctttttatctctcttggggaattggagctgcatgcgaattgagcgacagcaaaacgaactgcaagtcattgagaggagagcaaaaactcgagagcaagccaaagatggcgcaatctggggagagcgaaataaagctaaaatatgcatgttggagaaaaaatgccgcccatgtcgccaaaatgcgccacacgcagagtgagcgggcggaggtgggagtaatggaaagggcgatgagggaacgattagcttgaagagagagaacaacaaatgaatgtgctgcaacgttagttcaggtgagcgagttagagagagagttgttgttttttgattgtaatagctcgc

**ttggtggtg**ggtccacattcacatctccctctcccactctttctccccgaaagagagagcggga

**ci-200** ci-160

gcgaaggggcacgaggggagcacgatgactatgcagttgcattcaatttgaatttccatggtgctgatgattcgagcgccaattttttcgaagagttcttatttgtttacttcgttgttgttgcctcaattggaaagggaaaatgtggaatgcggagaaacaccagaagcaaatgcatttccattcataaatccaaagaagttttaaagataacatgtcatttggcttaagttcgtggtgcacaaaaaagatcggtttgcggttgtcgcatgaaaatgagtttattccattggtatattattattcagaaattaaaaaaaaacttgtttagtctattttttttttttaaataaaaaaaaaaaattcttttataagtcgattttagagtaaatatttaaagactacgtctaataaacatataatttgttctgtgttttaatttgccggcaaaaacaaacctact**tgtgtggtc**ctcgcacactcataacccctcgcatatttgagattcatgg

**ci-9**

ggcaaagaggctgcaaaaacaatggaaagggaaaagcagaaacatcctgccgctcataatttagcatcggaacatgcaaaaacagacatcatcgcatggggcagcagcaacagccataaaaccaacaacacgagcaatgtaaagctaacaaatttgccaacagttcgcggcacggctacacacacacacatgcatgcgcagcctgccacgcacgcgcttcccccaaacaaatacacacacacacactgagacgaaagctccattgggcagcgctgccgacgctgaaggccgacatcggcagagctgaacgtttgggtaggg**gaccaccca**catcgcttggcggtttcagtttaatgaaggcagaaacaaatttatttt

**---a-aa-- ci-1^ko^**

**tgggtggtc**cacactgcagcgaaaataaactacagtggcaacaacaaaccagcagccaaggcac

**---t-tt-- ci-1^ko^**

tt**tgggtggtc**catgcaaaaaaaaaacaaattacggcatgcgaataacaatagaaattagcgct

**---t-tt-- ci-1^ko^**

ctcgtggcggagctatttgggtatattagagctacatattttatttgtttataaaaagtataaatgtaaacaatgagttccaagcattaagtccgtatgctcaacaattacattatcattattattatcacttaaatatttacaaaggatatttaaacagtaatagatatatattttatttcttaatttctgttaacatatgtatttacattggtagttattctttattttgcaacaagcattcataaattttatataacaaacttggtattttctcggaaaaactcctgaatcacccctcggtattttgtgcgttgagctatcgttaaagcagccctcgcagagagcgttctcaaaccaaaatggccgcacacgaaacaagagagcgagtgagagtagggagagcgtctgtgttgtgtgttgagtgtc**gcccacgca**cacaggcgca

**ci-63**

aaacagtgcacacagacgcccgctgggcaagagagagtgagagaGAGAAACAGCGGCGCGCG

**JB chr2R:4,531,613-4,537,090 5478 bp**

TACGTACTCTTATTACTCCACTCcacttcacttcgcggcagtgtaaataaagacgggtcatgggatggggcacgaagtggggttaaatggccaggggcattggggagtaacgaggcatacctgcgggccgtaaataagcaactcaatgagcagcgaacgccagcgcacgtgcctcataaaccaccggccgagtcaccagtatccatacactcacagaaaaatggtggaacaaattcgatttaaaatttatattgacatttatttagcacttcaagctacaatagaaagacgtgacacattaaaatatttcatgtatctttcttcaaataaataatagctacaaatttttgcctgtgtacttaagtcgtattcgagccgagttggagtcaagtcgaatcgaatcgagttgga**gaccaccca**ggtaacgatttgtgcagtcattccacga

**ci-1**

attcccagctgagggggcgtcgcgtcggttcttcgtggcttttgcgtccaattctgcagacggcgggcgctggggggcatttcgcatttcaccaggctaagtatgctaaggatggcaaacggtatccgtccgggaccattgtcaaagtccaaattatgcatgcgttttcagagctgggtatacatacatatatagccggacagctgcgggagtgaaaataaaaccacaggaattaatttcgttcaaaataaatagatgtacttgctgttttgaactttctaatcatctacctacagactttaagatttggaatttccataacaatgtattccctgttacgcattgtaagtttctcgctggacacagtgcaatattcttatgaccagcactgtacttatgtaaattccgccggtggttgtggttgtggtgtgcagggcaataaggttcggcgcataac**gcccacgca**gtcgcagtatcgtcatggtcgccgtaatcgcagttggggcagcggg

**ci-63**

cgaaattaatttgacagccaacgcaaaacgcatgcgtcgtcgacattcctcgccttca

**ccccaccca**cccactgatcgccgtctcttgcaccaccctgctccccggatatatccacgatctc

**ci-104** ci-102

cggggattcgacgacgacgacaggcagacccagtgacgttccgggcttatcgccttactcttatatctatcttaagcttgttgttggcccgcctccttcccgctcattgtcgttatcgccttgcagtcacattaacacatccgacttcaaaaagggttatgcgatgagcttaactgatgtcgacggagcgggaactacgggcaaattaatgggttggggatttgcagccagcgttgcaaagggaacggaatcgaagagaacgtagcaatgtcatcttaattttggttctcaatatgatctctttagtgtagagcagaaacccagattacattgcccatcaatcaatcttcaaaatgccttcaaaaaagtgaactgcaatagaatatatctggaataaacaccttggacttactgaatcgcttctcattctacttccaattaattcccacttcataaccctctgaaatctggacaagacacttcgattccagctaaaataaacctatggaggaaatgtcgccatgcgtgggttcgctcagtgatagccgaaaatccatccatctgtaccgcaaaaaatctattgaaaacaaaacccaaataacctaggattgtacactgtaaaaaaacgaacattttctattgaaaaagtttttgtttctataaatacaactgatgaaaggttttaaaaatgagctaacccaatttttcttatgccaaaatagtacaatacaaatcgtctccgattgattttttgaaatcgaagtatcccgaatttgttcagctttttgaccataatttttgccaagtgcactgcgagaagcagagctgaaagcatggcag**gaccacaca**agaagctgcaaaagccacacacaccgtcgaggggacgggggcagact

**ci-9**

gctcagttccccagttcctcagttggtgatgtcggttgccggtcgccggtcgccggttgccgttggacgtggcggttgattacatgtacaaacgaaccgagccgatccgatccgatccgagcccagttccaggcccagtccgatctggtcccccagatcgcagagccaaccgcaaaagccgacgccaagataacgagttaacccaaacccagaccgcaggcaccggggaatcgggatgacgggccatcgggacaaggggagtcgcccg**aaccacaca**cgccatgtcgctggggattcggaatcgcggcctacgtattacc

**ci-196**

ggtggcaaatagtaattattacattaaatgcaccgcaataatgcagccgcgacgtcaacatttagccataaaaattgcttgcactccgctgttggaaatgcagttaagcttaaagtggcggtgcaagaggtacaccgaaaataattacaaataccaaatgttatgatattatgatagattaaacctttaaattgatatatttatggcattactatacatcaagtagcgaatccgaatagaaatagaattgacttcactgaatcacgtgaaagatctctttaaatttttttttttatgtaaaccttgtgaatgggttgtggcggtggaaaagggggatctagagaaaacaaccagccgggtaatggtaatggcgctatgggccaatgacaaatgcaatagcgttggtttggtaatcggggagcgggacgagaggattgtgtatcagttggggtctagggaacgctgcactataaattaagacattatacgattaaaaacaaacagcctgtcgttaaagtcgccaaaggaggaaaagagagcggagagcggagcgagcagatcagggttagaatccagcttaagcaacgggttgatgggcgagaccaaccgattggaatcattcacattttatagtaatggtaagggtatgttgcttaatgttgagaatttagtaaaaattttaaatttatttaaatgacttttgccacaactctactcccttttccactaagtctgcctcccgaaaaagctaaaaaaaaatagtttttgaatgcggggttcttaggagctttgtagagctcttaacgcttataacatgaaaaatatttatatttggtttatcgctttaaacgatcctgaattgagaaatatagattgaaacagaattcattaccatttaagaatatcattatttatgggggagtaatgcgcctccgagtaggcaatgcttttcttgacattgttactaagaattgtgaatgatatttgggcgtggatcaacgccgattaaaagctgcttttgcttccaggcggccagagaagagatccaaacttcaactccagccataaaagcaacaacatttccgtctcccccttgtagctccccttcctccggctcttccactctccacgaaacggcaaatgaagctctcaaagcgaactgtgcttcgctggtggtccattggcagctgccgccacacaggcgctgcttttgtgtgtgtgtgaatatcaatcttgctctccctctctttttatctctcttggggaattggagctgcatgcgaattgagcgacagcaaaacgaactgcaagtcattgagaggagagcaaaaactcgagagcaagccaaagatggcgcaatctggggagagcgaaataaagctaaaatatgcatgttggagaaaaaatgccgcccatgtcgccaaaatgcgccacacgcagagtgagcgggcggaggtgggagtaatggaaagggcgatgagggaacgattagcttgaagagagagaacaacaaatgaatgtgctgcaacgttagttcaggtgagcgagttagagagagagttgttgttttttgattgtaatagctcgc

**ttggtggtg**ggtccacattcacatctccctctcccactctttctccccgaaagagagagcggga

**ci-200** ci-161

gcgaaggggcacgaggggagcacgatgactatgcagttgcattcaatttgaatttccatggtgctgatgattcgagcgccaattttttcgaagagttcttatttgtttacttcgttgttgttgcctcaattggaaagggaaaatgtggaatgcggagaaacaccagaagcaaatgcatttccattcataaatccaaagaagttttaaagataacatgtcatttggcttaagttcgtggtgcacaaaaaagatcggtttgcggttgtcgcatgaaaatgagtttattccattggtatattattattcagaaattaaaaaaaaacttgtttagtctattttttttttttaaataaaaaaaaaaaattcttttataagtcgattttagagtaaatatttaaagactacgtctaataaacatataatttgttctgtgttttaatttgccggcaaaaacaaacctact**tgtgtggtc**ctcgcacactcataacccctcgcatatttgagattcatgg

**ci-9**

ggcaaagaggctgcaaaaacaatggaaagggaaaagcagaaacatcctgccgctcataatttagcatcggaacatgcaaaaacagacatcatcgcatggggcagcagcaacagccataaaaccaacaacacgagcaatgtaaagctaacaaatttgccaacagttcgcggcacggctacacacacacacatgcatgcgcagcctgccacgcacgcgcttcccccaaacaaatacacacacacacactgagacgaaagctccattgggcagcgctgccgacgctgaaggccgacatcggcagagctgaacgtttgggtaggg**gaccaccca**catcgcttggcggtttcagtttaatgaaggcagaaacaaatttatttt

**---a-aa-- ci-1^ko^**

**tgggtggtc**cacactgcagcgaaaataaactacagtggcaacaacaaaccagcagccaaggcac

**---t-tt-- ci-1^ko^**

tt**tgggtggtc**catgcaaaaaaaaaacaaattacggcatgcgaataacaatagaaattagcgct

**---t-tt-- ci-1^ko^**

ctcgtggcggagctatttgggtatattagagctacatattttatttgtttataaaaagtataaatgtaaacaatgagttccaagcattaagtccgtatgctcaacaattacattatcattattattatcacttaaatatttacaaaggatatttaaacagtaatagatatatattttatttcttaatttctgttaacatatgtatttacattggtagttattctttattttgcaacaagcattcataaattttatataacaaacttggtattttctcggaaaaactcctgaatcacccctcggtattttgtgcgttgagctatcgttaaagcagccctcgcagagagcgttctcaaaccaaaatggccgcacacgaaacaagagagcgagtgagagtagggagagcgtctgtgttgtgtgttgagtgtc**gcccacgca**cacaggcgca

**ci-63**

aaacagtgcacacagacgcccgctgggcaagagagagtgagagaGAGAAACAGCGGCGCGCG
