# Supplementary material for: An ancient yet flexible cis-regulatory architecture allows localized Hedgehog tuning by patched/Ptch1
Source: eLife. 2016 May 5;5:e13550. doi: 10.7554/eLife.13550 (PMC4887206; doi:10.7554/eLife.13550)
Supplement: Supplementary file 2. — Sequence alignments were obtained from the UCSC Genome Browser, dm6 build. Selected conserved Tango/Spineless (Tgo/Ss) motifs are in gray; Distaless (Dll) in light blue; Engrailed (En) in green; Pannier (Pnr/dGATAe) in purple; Scalloped (Sd) in blue; Cubitus interruptus (Ci) in red; Odd-Skipped (Odd) in dark red; Forkhead (Fkh) in dark yellow; Sloppy-paired (Slp) in blue-green; Pleiohomeotic (Pho) in pink, GAGA Factor (GAF/Trl) in yellow. Blocks of sequence separated by line breaks are not necessarily contiguous. DOI: http://dx.doi.org/10.7554/eLife.13550.022 [file elife-13550-supp2.docx]

**Supplementary File 2**

**Sequence conservation of selected TF binding motifs in enhancers of *patched.*** Selected conserved Tango/Spineless (Tgo/Ss) motifs are in gray; Distaless (Dll) in light blue; Engrailed (En) in green; Pannier (Pnr/dGATAe) in purple; Scalloped (Sd) in blue; Cubitus interruptus (Ci) in red; Odd-Skipped (Odd) in dark red; Forkhead (Fkh) in dark yellow; Sloppy-paired (Slp) in blue-green; Pleiohomeotic (Pho) in pink, GAGA Factor (GAF/Trl) in yellow. Blocks of sequence separated by line breaks are not necessarily contiguous.

**Enhancer ZY**

D. melanogaster tct----------------tcgtgggtggcccttaat------------------tcgacgtca-

D. simulans tct----------------tcgtgggtggcccttaat------------------tcgacgtca-

D. sechellia tct----------------tcgtgggtggcccttaat------------------tcgacgtca-

D. yakuba tct----------------tcgtgggtggcccttaat------------------tcgacgtca-

D. erecta tct----------------tcgtgggtggcccctaat------------------tcgacgtca-

D. biarmipes tct----------------tcgtgggtggcccttaat------------------tcgtcgtca-

D. bipectinata tct----------------tcgtgggtggcccttaat------------------tcgtcgtcag

D. eugracilis tct----------------tcgtgggtggcccttaat------------------tcgtcgtca-

D. elegans tct----------------tcgtgggtggcccttaat------------------tcgtcgtca-

D. takahashii tct----------------tcgtgggtggcccttaat------------------tcgtcgtca-

D. rhopaloa tct----------------tcgtgggtggcccttaat------------------tcgtcgtca-

D. ficusphila tct----------------tcgtgggtggcccttaat------------------tcgtcgtca-

D. pseudoobscura tcttgctctccgctctgcgtcgtgggtggcatttgat------tcgtcgtcgtcgtcgtcgtca-

D. persimilis tcttgctctccgctctgcgtcgtgggtggcatttgattcgtcgtcgtcgtcgtcgtcgtcgtca-

D. miranda tcttgctctccgctctgcgtcgtgggtggcatttgat------tcgtcgtcgtcgtcgtcgtca-

D. willistoni tct----------------tcgtgggtggcctt----------------------tcgttgtca-

D. virilis tct----------------tcgtgggtggccttttat------------------tcgttgtt--

D. mojavensis -------------------tcgtgggtggtcttttat------------------tcgttgct--

D. melanogaster tcttcttgggtggtcca-----------------------tattagccggcctgct--------------

D. simulans tcttcttgggtggtcca-----------------------tattagccggcctgtt--------------

D. sechellia tcttcttgggtggtcca-----------------------tattagccggcctgct--------------

D. yakuba tcttcttgggtggtcca-----------------------tatttgccggcctgct--------------

D. erecta tcttcttgggtggtcca-----------------------tattagccgtcctgct--------------

D. biarmipes tcctcttgggtggtcca-----------------------tattagccggccagtt--------------

D. bipectinata tctgcttgggtggcctc-----------------------tattagccgtcccgct--------------

D. eugracilis tcttcttgggtggtcca-----------------------tattagctggccttct--------------

D. elegans tcttcttgggtggtctc-----------------------tattagccggcctgct--------------

D. kikkawai tctgcttgggtggtc-------------------------tattagccggcctgct--------------

D. takahashii tcctcttgggtggtcca-----------------------tattagccggcctgtt--------------

D. rhopaloa tcttcttgggtggtctc-----------------------tattagccggcctgct--------------

D. ficusphila tcttcttgggtggtctc-----------------------tattagccggcctgct--------------

D. pseudoobscura tcatcatgggtggtc-------------------------tattagcccg--------------------

D. persimilis tcgtcatgggtggtc-------------------------tattagcccg--------------------

D. miranda tcgtcatgggtggtc-------------------------tattagcccg--------------------

D. willistoni tcgtcatgggtggtc-------------------------tattagctgcctcgcttctctatctttctc

D. virilis tcgtcttgggtggtctatctcttgccagtgtgtgtctctctgt---ctcgctcact--------------

D. mojavensis tcgccttgggtggtccaactcttggcagtgtgtggctctatat-agctcgctctcg--------------

D. melanogaster attgatcatt-ttctcgtttgatagtgcgtgacg--t---ctctgggcag------------------cc

D. simulans attgatcatt-ttctcgtttgatagtgcgtgacg--t---ctctgggcag------------------cc

D. sechellia attgatcatt-ttctcgtttgatagtgcgtgacg--t---ctctgggcag------------------cc

D. yakuba attgatcatt-ttctcgtttgatagtgcgtgacg--t---ctctgggcag------------------cc

D. erecta attgatcatt-ttctcgtttgatagtgcgtgacg--t---ctctgggcag------------------cc

D. biarmipes attgatcatt-ttctcgtttgatagtgcgtgacg--t---ctctgttcgg------------------ct

D. bipectinata attgatcattcccctcgtttgatagtgcgtgacgt-t---ctccagtact------------------cc

D. eugracilis attgatcatt-ttctcgtttgatagtgcgtgacg--tcgcctctggccga------------------ct

D. elegans attgatcatt-ttctcgtttgatagtgcgtgacg--t---ctctgagtaga-----------------cc

D. kikkawai attgatcatt-ctctcgtttgatagtgcgtgacg--a---cgcttgagagggctgccagaggagccgtcc

D. takahashii attgatcatt-ttctcgtttgatagtgcgtgacg--t---ctcgggtcgg------------------ct

D. rhopaloa attgatcatt-ttctcgtttgatagtgcgtgacg--t---ctctgggtagg-----------------cc

D. ficusphila attgatcatt-ttctcgtttgatagtgcgtgacgtct---ctctggacag------------------cc

D. pseudoobscura attgatcatt-ctctcgtttgatagtgcgtgacg--t---tttagtgttg------------------ct

D. persimilis attgatcatt-ctctcgtttgatagtgcgtgacg--t---tttagtgttg------------------ct

D. miranda attgatcatt-ctctcgtttgatagtgcgtgacg--t---tttagtgttg------------------ct

D. willistoni attgatcatt-ttcttgtttgatagtgcgtgacg--t---tttcaactctcact--------------ct

D. virilis attgatcatt-ttcttgtttgatagtgcgtgacg--t---tttcgtaatg------------------ct

D. mojavensis attgatcatt-ttctcgtttaatagtgcgtgacg--t---tttcataatg------------------ct

D. melanogaster cttctt-----ctgc------tgctcactcgc-----gctcatttattcgagcataaaattagcaattat

D. simulans cttctt-----ctgc------tgctcactcgc-----gctcatttattcgagcataaaattagcaattat

D. sechellia cttctt-----ctgc------tgctcactcgc-----gctcatttattcgagcataaaattagcaattat

D. yakuba cttctt-----ct--------cactccctcgc-----gctcatttgttcgagcataaaattagcaattac

D. erecta cttctg-----ct--------ccctcgctcgc-----gctcatttattcgagcataaaattagcaattat

D. biarmipes cttcgt-----ctgc------tgctcactcgc-----gctcatttattcgagcataaaattagcaattat

D. bipectinata cttcgc-----ctgc------tcttcattcgc-----gctcatttattcgagcataaaattagcaattat

D. eugracilis cttcgt-----ctccgctgctggctcactcgc-----gctcatttattcgagcataaaattagcaattat

D. elegans cttcgt-----ctgc------gtctcactcac-----gctcatttattcgagcataaaattagcaattat

D. kikkawai ctttgc-----ctgcccttc-gtctcgctagc-----tctcatttattcgagcataaaattagcaattat

D. takahashii cttcgt-----ctcc------tgctcactcgc-----gctcatttattcgagcataaaattagcaattat

D. rhopaloa cttcgt-----ctgc------ggctcactcgc-----gctcatttattcgagcataaaattagcaattat

D. ficusphila cttcgt-----ctgc------tgctcactcgc-----gctcatttattcgagcataaaattagcaattat

D. pseudoobscura cttctttactcttgg------ggcacactcacgctcagctcatttattcgagcataaaattagcaattat

D. persimilis cttcttaatccttgg------ggcacactcacgctcagctcatttattcgagcataaaattagcaattat

D. miranda cttctttactcttgg------ggcacactcacgctcagctcatttattcgagcataaaattagcaattat

D. willistoni ctactt---------------ggggcactcag-----gctcatttattcgagcataaaattagcaattat

D. virilis cttcaa-------------------------------cgttttttattcgagcataaaattagcaattat

D. mojavensis cttttg-------------------------------agttttttattcgagcataaaattagcaattat

**Enhancer YU**

D. melanogaster tgttggca-acaatta-ttgcgttgttgacagcaagatagatagcaatcatttgc

D. simulans tgttggca-acaatta-ttgcgttgttgacagcaagatagatagcaatcatttgc

D. sechellia tgttggca-acaatta-ttgcgttgttgacagcaagatagatagcaatcatttgc

D. yakuba tgttggca-acaatta-ctgtgttgttgacagcaagatagatagcaatcatttgc

D. erecta tgtt-gca-acaatta-ttgtgttgttgacagcaagatagatagcaatcatttgc

D. biarmipes tgtttgca-acaatta-ttgtgttgttgacagcaagatagataggaataatttgc

D. bipectinata ------ct-ac-atta-ttttgatgttgacagcaagatagatagcaataatttgc

D. eugracilis tgttggca-acaatta-ttgtgttgttgacagcaagatagatagcaataatttgc

D. elegans -----gca-acaatta-ttgtgatgttgacagccagatagatagcaataatttgc

D. kikkawai tgtcggca-acaatta-ttgtgttgttgacggccagatagatagcaataatttgc

D. takahashii tgttggca-acaatta-ttgtgttgttgacagcaagatagatagcaataatttgc

D. rhopaloa tgttggca-acaatta-ttgtgttgttgacagcaagatagatagcaataatttgc

D. ficusphila tgttggca-acaatta-ttgtgttgttgacagcaagatagatagcaacaatttgc

D. pseudoobscura tgttacca-acaatta-ttccgatgttgacagcaagatagatagcaataatttgc

D. persimilis tgttacca-acaatta-ttccgatgttgacagcaagatagatagcaataatttgc

D. miranda tgttacca-acaatta-ttccgatgttgacagcaagatagatagcaataatttgc

D. virilis tgttgcca-acaattatttgtgttgttgacagcaagacagatagcaacagcagca

D. mojavensis tgttgcca-acaattatttgtgttgttgacagcaagacagatagcaacagcaaca

D. melanogaster gtcccatcgtataattgcatatagcgccagtggaagttccggtcggttggc--tttttt

D. simulans gtccgatcgtataattgcatatagcgccagtggaagttccggtcggttggc--tttttt

D. sechellia gtccgatcgtataattgcatatagcgccagtggaagttccggtcggttggc--tttttt

D. yakuba -tgcgatcgtataattgcatatagcgccagtggaagttccggtcggttggc--tttttg

D. erecta ---cgatcggataattgcatatagcgccagtggaagttccggtcggttggc--tttttt

D. biarmipes ------tcgtataattgcatatagcgccagttgaagttccggtcggttggg--tttttt

D. bipectinata ------tcttataattgcatatagcgccagttgaagttccggtcggttgag--tttttg

D. eugracilis ------tcgtataattgcatatagcgccagttgaagttccggtcggttggg--tttttt

D. elegans ------tcgtataattgcatatagcgccagttgaagttccggtcggttggc--tttttt

D. kikkawai ------tcttataattgcatatagcgccagttgaagttccggtcggttggg--tttctt

D. takahashii ------tcgtataattgcatatagcgccagttgaagttccggtcggttggg--tttttt

D. rhopaloa ------tcgtataattgcatatagcgccagttgaagttccggtcggttggg--tttttt

D. ficusphila ------tcgtataattgcatatagcgccagttgaagttccggtccgttggg--tttttt

D. pseudoobscura ------tcttataattgcatatagcgccagttgcagttccggtcggttggg--tttttt

D. persimilis ------tcttataattgcatatagcgccagttgcagttccggtcggttggg--tttttt

D. miranda ------tcttataattgcatatagcgccagttgcagttccggtcggttgggtttttttt

D. willistoni ------ttttataattgcatatagcgccagttgcagttccggtccgatgag--tttttg

D. virilis ------gtttataattgcatatagcgccagttgcagttccggtccgttgag--tttttt

D. mojavensis ------gcttataattgcatatagcgccagttgcagttccggtcggttgag--tttttt

D. melanogaster tgttgctgtgtatgtgtgattggtgaatggcatgttgccacccaagttgcca-----------gttggct

D. simulans tgctgctgtgtatgtgtgattggtgaatggcatgttgccacccaagttgcca-----------gttggct

D. sechellia tgctgctgtgtatgtgtgattggtgaatggcatgttgccacccaagttgcca-----------gttggct

D. yakuba tgttgctgtgtatgtgtgattggtgaatggcatgttgccacccaagttgcca-----------gttggct

D. erecta tgtgcctgtgtatgtgtgattggtgaatggcatgttgccacccaagttgcca-----------gttggct

D. biarmipes tgtccctgtgtatgtgtgattggtgaatggcatgttgccacccaag------------------------

D. bipectinata tgtatacgagtatgtgtgattggtgaatgccatgttgccacccaagttgccaactggcaactggctggct

D. eugracilis tgctcctgtgtatgtgtgattggtgaatggcatgttgccacccaagttgcca-----------gttggcc

D. elegans tgttcttgtgtatgtgtgattggtgaatggcatgttgccacccaagttgcca-----------gttggct

D. kikkawai tgcccttgtgtatgtgtgattggtgaatggcatgttgccacccaagttgcca-----------gttggct

D. takahashii tgtccctgtgtatgtgtgattggtgaatggcatgttgccacccaagttgcca-----------gttggct

D. rhopaloa tgtgcttgtgtatgtgtgattggtgaatggcatgttgccacccaagttgcca-----------gttagct

D. ficusphila tgtgcttgtgtatgtgtgattggtgaatggcatgttgccacccaagttgcca-----------gttggct

D. pseudoobscura agtactggtatatgtgtgattggtgaatggcatgttgccacccaagttgccaactgtgcctcggttggct

D. persimilis agaactggtatatgtgtgattggtgaatggcatgttgccacccaagttgccaactgtgcctcggttggct

D. miranda agtactggtatatgtgtgattggtgaatggcatgttgccacccaagttgccaactgtgcctcggttggct

D. virilis tgtttgtgtgtgtgtgggattggtgaatggcatgttgccacccaagttgccaactgtgtcttggttggct

D. mojavensis tgtgtgtgtatgagtgggattggtgaatggcatgttgccacccaagttgccaactgtgtcttggttggct

**Enhancer VT**

D. melanogaster tggtgaatggcatgttgccacccaagttgcca-----------g

D. simulans tggtgaatggcatgttgccacccaagttgcca-----------g

D. sechellia tggtgaatggcatgttgccacccaagttgcca-----------g

D. yakuba tggtgaatggcatgttgccacccaagttgcca-----------g

D. erecta tggtgaatggcatgttgccacccaagttgcca-----------g

D. biarmipes tggtgaatggcatgttgccacccaag------------------

D. bipectinata tggtgaatgccatgttgccacccaagttgccaactggcaactgg

D. eugracilis tggtgaatggcatgttgccacccaagttgcca-----------g

D. elegans tggtgaatggcatgttgccacccaagttgcca-----------g

D. kikkawai tggtgaatggcatgttgccacccaagttgcca-----------g

D. takahashii tggtgaatggcatgttgccacccaagttgcca-----------g

D. rhopaloa tggtgaatggcatgttgccacccaagttgcca-----------g

D. ficusphila tggtgaatggcatgttgccacccaagttgcca-----------g

D. pseudoobscura tggtgaatggcatgttgccacccaagttgccaactgtgcctcgg

D. persimilis tggtgaatggcatgttgccacccaagttgccaactgtgcctcgg

D. miranda tggtgaatggcatgttgccacccaagttgccaactgtgcctcgg

D. virilis tggtgaatggcatgttgccacccaagttgccaactgtgtcttgg

D. mojavensis tggtgaatggcatgttgccacccaagttgccaactgtgtcttgg

D. melanogaster gcatc------aaacggtagc-tcttcttaattacaacaaccaa-------aaca-------acagc---

D. simulans gcatc------aaacggtagc-tcttcttaattacaacaaccaa-------aaca-------acagc---

D. sechellia gcatc------aaacggtagc-tcttcttaattacaacaaccaa-------aaca-------acagc---

D. yakuba gcatc------aaacggtagc-tcttcttaattacaacaaccaa-------aaca-------acagc---

D. erecta gcatc------aaacggtagc-tcctcttaattacaacaaccaa-------aaca-------acagc---

D. biarmipes gcatc------aaacggtagc-tcttcttaattacaacaaccaa-------aacaacc----acagc---

D. bipectinata agtcccctccaaaacggtagccccctcttaattacaataacaac-------aaca-------aaaac---

D. eugracilis gcatc------aaacggtagc-tgctcttaattacaacaaccaaaaacaacaaca-------acaac---

D. elegans gcatc------aaacggtagc-tctccttaattacaacaaccga-------agca-------gcaacaa-

D. kikkawai gcatc------aaacggtagc-tcttcttaattacaacaacgac-------agca-------accacag-

D. takahashii gcatc------aaacggtagc-tcttcttaattacaacaaccaa-------aaca-------acaac---

D. rhopaloa gcatc------aaacggtagc-tcttcttaattacaacaactaa-------aaca-------acagt---

D. ficusphila gcatc------aaacggtagc-tcttcttaattacaacaaccaa-------aaca-------acaac---

D. pseudoobscura acatc------aaacggtagc-tcctcttaattacaacaacacc-------aacatccagaagcaacaag

D. persimilis acatc------aaacggtagc-tcctcttaattacaacaacacc-------aacatccagaagcaacaag

D. miranda acatc------aaacggtagc-tcctcttaattacaacaacacc-------aacatccagaagcaacaag

D. willistoni ggagc------aaacggtagc-tgttcttaattacaacagcaac-------aaca-------ataacaac

D. virilis gcgcc------aaacggtagc-cattcttaattacaacaacaac-------aaca-------acaac---

D. mojavensis acaac------aac---------------aacagcaacaacaac-------atca-------gcagc---

D. melanogaster aatcggaaacggaaatcgctaatg-aaa--aatttccatatttatggtaacgacaaag--agccgc-ttc

D. simulans aatcggaaacggaaatcgctaatg-aaa--aatttccatatttatggtaacgacaaag--agccgc-ttc

D. sechellia aatcggaaacggaaatcgctaatg-aaa--aatttccatatttatggtaacgacaaag--agccgc-ttc

D. yakuba aatcggaaacggaaatcgctaatg-aaa--aatttccatatttatggta-------------------tc

D. erecta aatcggaaacggaaatcgctaatg-aaa--aatttccatatttatggtaacgacaaag--agccgc-gtc

D. biarmipes aatcggaaacggaaatcgctaatg-aaa--aatctccatatttatggtggcgataaag--agccac----

D. bipectinata aatcggaaacggaaatcactaatg-aaa--aatttccatatttatggtggcaataaag--agtc-----t

D. eugracilis aatcggaaacggaaatcgctaatg-aaa--aatttccatatttatggtggcgataaag--agccac----

D. elegans aatcggaaacggaaatcgctaatg-aaa--aatttccatatttatggtggcgataaag--agccca----

D. kikkawai aatcggaaacggaaatcgctaatg-aaa--aatttccatatttatggtggcgataaag--cgatac-aac

D. takahashii aatcggaaacggaaatcgctaatg-aaa--aatctccatatttatggcggcgataaag--agccac----

D. rhopaloa aatcggaaacggaaatcgctaatg-aaa--aatttccatatttatggcggcgataaag--agccc-----

D. ficusphila aatcggaaacggaaatcgctaatg-aaa--aatttccatatttatggcggcgataaag--agccgcagcc

D. pseudoobscura aatcggaaacggaaatcgctaatg-aaa--aatttccatatttatgcagccgataaag--cgctac----

D. persimilis aatcggaaacggaaatcgctaatg-aaa--aatttccatatttatgcagccgataaag--cgctac----

D. miranda aatcggaaacggaaatcgctaatg-aaa--aatttccatatttatgcagccgataaag--cgctac----

D. willistoni aatcggaaacggaaatcgctaatgaaaa--aatttccatatttatgc--------atg--tgcc------

D. virilis aatcggaaacggatatcgctaatg-aaa--aatttccatatttatg-tggcgataaaagcagc-ac----

D. mojavensis aatcggaaacggatatcgctaatg-aaacgaatttccatatttatg-tgttaataaaaccagcaac----

D. melanogaster tacgggctgcaattaattccaacgaa-attta--attag-aat

D. simulans tacgggctgcaattaattccaacgaa-attta--attag-aat

D. sechellia tacgggctgcaattaattccaacgaa-attta--attag-aat

D. yakuba tacggactgcaattaattccaacgaa-attta--attag-aat

D. erecta tacgggctgcaattaattccaaccaa-attta--attag-aat

D. biarmipes cacgggctgcaattaattccaccgaa-attta--attag-aat

D. bipectinata tatgtgttgcaattaattccaatgaa-attta--attag-aat

D. eugracilis tacgggctgcaattaattccaacgaa-attta--attag-aat

D. elegans tatgggctgcaattaattccaacaaa-attta--attag-aat

D. kikkawai tacggactgcaattaattccaacgaa-attta--attag-aat

D. takahashii tacgggctgcaattaattccaacgaa-attta--attag-aat

D. rhopaloa tatgggctgcaattaattccaacgaa-attta--attag-aat

D. ficusphila tatgggctgcaattaatcccaacgaa-attta--attag-aat

D. pseudoobscura tatgtgtcgcaattaatttcaacgaa-attta--attag-aat

D. persimilis tatgtgtcgcaattaatttcaacgaa-attta--attag-aat

D. miranda tatgtgtcgcaattaattttaacgaa-attta--attag-aat

D. willistoni catag--cgcaattaatttcatcaaa-tttca--ttcagaaat

D. virilis tgtgtgatgcaattaatttcatcgaatatttgttgttgg-aat

**Enhancer RP**

D. melanogaster gcccgtgcccaatggccataattaattgct

D. simulans gcccgtgcccaatggccataattaattgct

D. sechellia gcccgtgcccaatggccataattaattgct

D. yakuba gcccgtgcccaatggccataattaattgct

D. erecta gcccgtgcccaatggccataattaattgct

D. biarmipes gcccgtgcccaatggccataattaattgct

D. bipectinata gtccaaacccgatggctataattaattgct

D. eugracilis gcccgtgcccaatggccataattaattgct

D. elegans gcccgtgcccaatggccataattaattgct

D. kikkawai gtccgtgcccaatggctataattaattgct

D. takahashii gcccgtgcccaatggccataattaattgct

D. rhopaloa gcccgtgcccaatggccataattaattgct

D. ficusphila gcccgtgcccaatggccataattaattgct

D. pseudoobscura gtccctgcccaatggccataattaatcgct

D. persimilis gtccctgcccaatggccataattaatcgct

D. miranda gtccctgcccaatggccataattaatcgct

D. willistoni gttcatgcccattggctataattaattggt

D. virilis tccaatactcaatgaccataattaattggt

D. melanogaster ag--tcgtgctaaggtcaaacaaacagcc

D. yakuba ag--tcgtgctaaggtcaaacacacagcc

D. erecta ag--tcgtgctaaggtcaaacacacagcc

D. biarmipes aa--ccgtgctaaggtcaaacacacagcc

D. bipectinata aa--ccatgctaaggtcaaacacagagcc

D. eugracilis aa--ccgtgctaaggtcaaacacacagcc

D. elegans aa--tcatgctaaggtcaaacacacagcc

D. kikkawai aa--gcatgctaaggtcaaacacagagcc

D. takahashii aa--tcgtgctaaggtcaaacacacagcc

D. rhopaloa aa--tcgtgctaaggtcaaacac--agcc

D. ficusphila aa--tcgtgctaaggtcaaacacacagcc

D. pseudoobscura aa--ccatgctaaggtcaaacacacagcc

D. persimilis aa--ccatgctaaggtcaaacacacagcc

D. miranda aa--ccatgctaaggtcaaacacacagcc

D. willistoni gatttcaggcaaaggtcaaacacgtgcta

D. virilis gc--acatgctgaggtcaaacgcacagag

D. mojavensis gc--acatgctgaggtcaagcacacacac

D. melanogaster agccgtgacag-acttgtgggcaaacagaaacca

D. sechellia agccgtgacag-acttgtgggcaaacagacacaa

D. yakuba agccgtgacag-acttgtgggcaaacagacacca

D. erecta ggccgtaacag-acttgtgggcaaacagacacca

D. biarmipes agccgtggcag-acttgtgggtaaacagactcca

D. bipectinata ggccgtgacag-acttgtgggtaaacagacacca

D. eugracilis agccgtgacag-acttgtgggtaaacagacacca

D. elegans agccgtgacag-acttgtgggtaaacagaaacca

D. kikkawai ggccgtgacag-acttgtgggtaaacagacacca

D. takahashii agccgtgacag-acttgtgggtaaacagaaacca

D. rhopaloa agccgtgacag-acttgtgggtaaacagacacca

D. ficusphila agccgtgactg-acttgtgggtaaacagaaacca

D. pseudoobscura ggccgtgacag-acttgtgggtaaacagaaacca

D. persimilis ggccgtgacag-acttgtgggtaaacagaaacca

D. miranda ggccgtgacag-acttgtgggtaaacagaaacca

D. willistoni aaccgtgacaacaattgtgggtaaacagaaacca

D. virilis -accatgacgt-tgttgcgggtaaataaaaacca

D. mojavensis -accatgacca-tgttgtgggtaaatagaactca

D. melanogaster ttc--acacaaccgcaaagcagttgaacaaacaatgat------cc------g

D. simulans ttc--acacaaccgcaaagcagttgaacaaacaatgat------cc------a

D. sechellia ttc--acacaaccgcaaagcagttgaacaaacaatgat------cc------g

D. yakuba ttt--acacaaccgcaaggcagttgaacaaacaatgat------cc------g

D. erecta ttt--acacaaccgcaaagcagttgaacaaaca--------------------

D. biarmipes ttc--acacaaccgcaaagcagttgaacaaacaatgat------ct------g

D. bipectinata tcc--acacaaccgcaaaacagttgaacaaacaatgat------cc------g

D. eugracilis ttc--acacaaccgcaaagcagttgaacaaacaatgat------ct------g

D. elegans ttc--acacaaccgcaaagcagttgaacaaacaatgat------ct------g

D. kikkawai ttt--acacaaccgcagaacagttgagcaaacaatgat------ccacaacag

D. takahashii ttc--acacaaccgcaaagcagttgaacaaacaatgat------cc------g

D. rhopaloa tcc--acacaaccgcaaagcagttgaacaaacaatgct------ct------g

D. ficusphila ttc--acacaaccgcaaagcagttgaacaaacaatgct------cc------g

D. pseudoobscura ttt--acacaaccgcaaaacagttggacaaacaatgat------cc------g

D. persimilis ttt--acacaaccgcaaaacagttggacaaacaatgat------cc------g

D. miranda ttt--acacaaccgcaaaacagttggacaaacaatgat------cc------g

D. willistoni ttt--acacaaccgcaaaacatttgtacaaacaatgat------cg------g

D. virilis ttt--acacagccgcag------------aacaatgat----ctct------t

D. mojavensis tttacacacaaccacaa-------gaac-aacaatgatttccctct------

**Enhancer PN**

D. melanogaster c--accacca------------------------c---------------------ggcgttgataattg

D. simulans ----ccacca------------------------c---------------------ggcgttgataattg

D. sechellia ----ccacca------------------------c---------------------ggcgttgataattg

D. yakuba ----ccgcca------------------------c---------------------ggcgttgataattg

D. erecta ----ccgccg------------------------c---------------------ggcgttgataattg

D. biarmipes ----ccgccc------------------------c---------------------ggcgttgataattg

D. bipectinata ----ctactactcgcttttcattccatcgcatggc---------------------agcgttgataattg

D. eugracilis ----ccgcca------------------------t---------------------ggcgttgataattg

D. elegans ----ccacca------------------------c---------------------ggcgttgataattg

D. kikkawai ttggccgcct------------------------c---------------------agcgttgataattg

D. takahashii ----ccacca------------------------c---------------------ggcgttgataattg

D. rhopaloa ----ccgctg------------------------c---------------------ggcgttgataattg

D. ficusphila ----ccggct------------------------c---------------------ggcgttgataattg

D. pseudoobscura ----ctactc------------------------c-------------------cgggcgttgataattg

D. persimilis ----ctactc------------------------c-------------------cgggcgttgataattg

D. miranda ----ctactc------------------------c-------------------cgggcgttgataattg

D. willistoni ----ttgcct------------------------c-----------ggcatctgtctctctggccaatgg

D. mojavensis ---tctatcc------------------------agacgagagacaagcgtt----------aataatca

D. virilis ======================================================================

D. melanogaster cgggtgg-----cc-gtgactgaggcgataact-------------------------------------

D. simulans cgggtgg-----cc-gtgactgaggcgataact-------------------------------------

D. sechellia cgggtgg-----cc-gtgactgaggcgataact-------------------------------------

D. yakuba cgggtgg-----cc-gtgactgaggcgataact-------------------------------------

D. erecta cgggtgg-----cc-gtgactgaggcgataact-------------------------------------

D. biarmipes cgggtgg-----cc-gtcacgaaggcgataagc----gatg--gcaaat------------------gct

D. bipectinata cgggtgg-----ccggtatcgaaggcgataact----gata--ccaaat------------------gcc

D. eugracilis cgggtgg-----cc-gtaacggatctgataacctgatgatg--gcgaat------------------gta

D. elegans cgggtgg-----cc-gtaacgaaggcgataacc----gatg--gcaaat------------------gct

D. kikkawai cgggtgg--cgacc-ataacgaaggtgataact----ga-----------------------------ta

D. takahashii cgggtgg-----cc-gtaactaaggcgataacc----gatg--gcgaag------------------gct

D. rhopaloa cgggtgg-----cc-gtaacgaaggcgataacc----gatgctgcaaat------------------gcc

D. ficusphila cgggtgg-----cc-gtaacgaaggcgataacc----gatg--gcacatcctgcacctccacctccacct

D. pseudoobscura cgggtgg-----cc-gtaactgaagtgataagt----gaca--gccatt------------------gcc

D. persimilis cgggtgg-----cc-gtaactgaagtgataagt----gaca--gccatt------------------gcc

D. miranda cgggtgg-----cc-gagactgaagtgataagt----gaca--gccagt------------------gcc

D. willistoni cgggtgg-----cc-gtaactgaagtgataagt----gt----tctaat------------------gct

D. mojavensis cgggtggtataccc-atatccaa-gtgccgaac----tctc--tcagat------------------ctt

D. virilis ======================================================================

D. melanogaster a-aacatcttgt--gactatcatc----------------aatcgg--ggagcgca-------ccttgat

D. simulans a-aacatcgtac--gactatcttc----------------aatcgg--gaagcgca-------ccttgat

D. sechellia a-aacatcgtac--gactatcttc----------------aatcgg--cgagcgca-------ccttgat

D. yakuba a-aacatcgtac--gattatcttc----------------cattgg--ggagcgca-------ccttgat

D. erecta a-aactgcgcac--gattatcttc----------------catcag--ggaggatg-------gcttgat

D. biarmipes ataacatcttac--gattatctgc----------------aatcag--ggagccca-------ccttgat

D. bipectinata a-aatatcatac--gattatctgc----------------aatcagtccgagtgtgtgagttcccttgat

D. eugracilis a-aacatcttac--gattatctgc----------------catcagt-ggggacca-------ccttgat

D. elegans a-aacatcttac--gattatctgc----------------aatcag--cgggacca-------ccttgat

D. kikkawai a-aacattgttcgagattacctac----------------aatcag--cgagccca-------ccttgat

D. takahashii aaaacatcttac--gattatctgc----------------aatcag--agaacaca-------gcttgat

D. rhopaloa a-aacatcttac--gattatctgc----------------aatcag--cgggccca-------ccttgat

D. ficusphila a-aacatcttac--gattatctgc----------------catcag--cgagccca-------ccttgat

D. pseudoobscura a-aacattgtac--aattatctgcaacttccatca-----gatcggcctggttctc-------ccttgat

D. persimilis a-aacattgtac--aattatctgcaacttccatca-----gatcggcctggttctc-------ccttgat

D. miranda a-aacattgtac--aattatctgcaacttccatcagatcggatcggcctggttctc-------ccttgat

D. willistoni a-aaaa--gttc--aatgaactgg-----taaaaa-----aatggg--aaagaata-------tttttat

D. virilis a-aatgttcgct--ggcaatcaac----------------agtcaa--ctgttggc-------ccttgga

**Enhancer LJ**

D. melanogaster aatgtaaacaaatgatatacggtcg------cagggc

D. simulans aatgtaaacaaatgatatacggtcg------cagggc

D. sechellia aatgtaaacaaatgatatacggtcg------cagggc

D. yakuba aatgtaaacaaatgatatacggtcg------cagggg

D. erecta aatgtaaacaaatgatatacggtcg------caggga

D. biarmipes aatgtaaacaaatgatatacggtcg------cagggc

D. bipectinata aatgtaaacaaatgatatacggtcggtggcccaggtc

D. eugracilis aatgtaaacaaatgatatacggtcg------cagggc

D. elegans aatgtaaacaaatgatatacggtca------cagggt

D. kikkawai aatgtaaacaaatgatatacggtcg------cagggc

D. takahashii aatgtaaacaaatgatatacggtcg------cagggc

D. rhopaloa aatgtaaacaaatgatatacggtcg------cagggc

D. ficusphila aatgtaaacaaatgatatacggtcg------cagggc

D. pseudoobscura aatgtaaacaaatgatatacggtca------cagggc

D. persimilis aatgtaaacaaatgatatacggtca------cagggc

D. miranda aatgtaaacaaatgatatacggtca------cagggc

D. willistoni aatgtaaacaaatgatatacggtca------cagagc

D. virilis aatgtaaacaaatgatatacggtca------cagtcc

D. mojavensis aatgtaaacaaatgatatacggtca------cagttc

D. melanogaster cacttcacttcgcggcagtgtaaataaa--------------------gacgg-g-----tcat------

D. simulans cactccacttcgcggcagtgtaaataaa--------------------gacgg-g-----tcgt------

D. sechellia cactccacttcgcggcagtgtaaataaa--------------------gacgg-g-----tcgt------

D. yakuba cacttcacttcacggcagtgtaaataaa--------------------gacgg-g-----tcgt------

D. erecta cacttcacttcacggcagtgtaaataaa--------------------gacgg-g-----tcgt------

D. biarmipes tacttcacttcacggcagtgtaaataaa--------------------gacgg-g------ctt------

D. bipectinata tacttcacttcacgccagtgtaaataat--------------------gcctc-g----tttgt------

D. eugracilis cacttcacttcacggcagtgtaaataaa--------------------gacgg-c-----ttgttgactg

D. elegans tacttcacttcacggcagtgtaaataaa--------------------gatgg-g-----gcga------

D. kikkawai cacttcacttcacggcaatgtaaataaa--------------------gtagg-g-----ttat------

D. takahashii cacttcacttcacggcagtgtaaataaa--------------------gacgg-g----gccgt------

D. rhopaloa cacttcacttcacggcagtgtaaataaa--------------------gatgg-g-----gcga------

D. ficusphila cacttcacttcacggcagtgtaaataaa--------------------gacgatg-----gtgt------

D. pseudoobscura cacttgacttcacaccaatataaataca--------------------gccgt-a-----acgt------

D. persimilis cacttgacttcacaccaatataaataca--------------------gccgt-a-----gcgt------

D. miranda cacttgacttcacaccaatacaaataca--------------------gccgt-a-----gcgt------

D. willistoni ctcttcacttcacacc------aataaa--------------------ga----g-----tcgg------

D. virilis -acttcacttcacaccaataaaaatacaacaaaaataaactgaacacgggagt-g-----gtga------

D. mojavensis cacttcacttcacacccataaaaaacaaagagaaggcagctcgagtgggacgt-gtgtgtgtgg------

D. melanogaster tggggagtaacgaggca------tacctgcgggccgtaaataagcaa

D. simulans tggggagtaacgaggca------taccttcgggccgtaaataagcaa

D. sechellia tggggagtaac-aggca------taccttcgggccgtaaataagcaa

D. yakuba tgcggagtaacgaggcatactcgtaccttcgggccgtaaataagcaa

D. erecta tggggagtaacgaggca------taccttcgggccgtaaataagcaa

D. biarmipes tggggagtaacgaggca------tacctccgggccgtaaataagcaa

D. bipectinata ggggcagcaacgagaca------tacctttgagccgtaaataagcaa

D. eugracilis tggggagtaacgaggca------taccttcgggccgtaaataagcaa

D. elegans tggggagtaacgaggca------taccttcgggccgtaaataagcaa

D. kikkawai tggggagtaacgaggca------taccttcgggccgtaaataagcaa

D. takahashii tggggagtaacgaggca------taccttcgggccgtaaataagcaa

D. rhopaloa tggggagtaacgaggca------taccttcgggccgtaaataagcaa

D. ficusphila tggggagtaacgaggca------taccttcggaccgtaaataagcaa

D. pseudoobscura tggggg--aacgaa--a------taccttcaagccgtaaataagcaa

D. persimilis tggggg--aacgaa--a------taccttcaagccgtaaataagcaa

D. miranda tggggg--aacgaa--a------taccttcaagccgtaaataagcaa

D. willistoni --------atcgaa--a------taccttcgagccgtaaataagcaa

D. virilis --ggccacaccga---a------taccttaaagccgtaaataagcaa

D. mojavensis -tgaccacaccga---a------taccttcaagccgtaaataagcaa

**Enhancer JB**

D. melanogaster gagaccacccaggtaacgatttgtgcagtcattccacgaattcccagc

D. simulans gagaccacccaggtaacgatttgtgcagtcattccacgaattcccagc

D. sechellia gagaccacccaggtaacgatttgtgcagtcattccacgaattcccagc

D. yakuba gagaccacccaggtaacgatttgtgcagtcattccacgaattcccagc

D. erecta gagaccacccaggtaacgatttgtgcagtcattccacgaattcccagc

D. biarmipes gagaccacccaggtaacgatttgtgcagtcattccacgaattcccagc

D. bipectinata gagaccacccaggtaacgatttgtgcagtcattccacgaattcccaac

D. eugracilis gagaccacccaggtaacgatttgtgcagtcattccacgaattcccagc

D. elegans gagaccacccaggtaacgatttgtgcagtcattccacgaattcccaga

D. kikkawai gagaccacccaggtaacgatttgtgcagtcattccacgaattcccaac

D. takahashii gagaccacccaggtaacgatttgtgcagtcattccacgaattcccagc

D. rhopaloa gagaccacccaggtaacgatttgtgcagtcattccacgaattcccaga

D. ficusphila gagaccacccaggtaacgatttgtgcagtcattccacgaattcccagc

D. pseudoobscura gggaccacccaggtaacgatttgtgcagtcattccacgaattctcgga

D. persimilis gggaccacccaggtaacgatttgtgcagtcattccacgaattctcgga

D. miranda gggaccacccaggtaacgatttgtgcagtcattccacgaattctcgga

D. willistoni gag--cacaggtataacgataagagaagt-------------------

D. virilis gagaccacccaggcgtcgatttgtgcagtcattccacgaattcttggc

D. mojavensis gagaccacccaggcgacgatttgtgcagtcattccacgaattcttggc

D. melanogaster cgaaattaatttgacagccaacgcaaaacgcatgcgtcgtcgacattcctcgcc----------------

D. simulans cgaaattaatttgacagccaacgcaaaacgcatgcgtcgtcgacattcctcgcc----------------

D. sechellia cgaaattaatttgacagccatcgcaaaacgcatgcgtcgtcgacattcctcgcc----------------

D. yakuba tgaaattaatttgacagccaacgcaaaacgcatgcgtcgtcggcattcctcgccagcactctaccccctt

D. erecta tgaaattaatttgacagccaacgcaaaacgcatg---cgtcgacattcttcgcc----------------

D. biarmipes cgaaattaatttgacagccaacgcaaaacgcatgcttcgtcaacattcctcgcc----------------

D. bipectinata cgaaattaatttgacagccaacgcaaaacgcatgcgtcttcgacattcttcact----------------

D. eugracilis cgaaattaatttgacagccaacgcaaaacgcatgcgtcgtcaacattcttcgcc----------------

D. elegans cgaaattaatttgacagccaacgcaaaacgcatgcgtcgtcaacattcctcgcc----------------

D. kikkawai cgaaattaatttgacagccaacgcaaaacgcatgcgtcgtcaacattcctggcc----------------

D. takahashii cgaaattaatttgacagccaacgcaaaacgcatgcttcgtcaacattcctcgcc----------------

D. rhopaloa cgaaattaatttgacagccaacgcaaaacgcatgcgtcgtcaacattcctcgcc----------------

D. ficusphila cgaaattaatttgacagccaacgcaaaacgcatgcgtcgtcaacattcctcgcc----------------

D. pseudoobscura cgaaattaatttgacagccaacgcaaaacgcatgcatcgtcaacattcttctgg----------------

D. persimilis cgaaattaatttgacagccaacgcaaaacgcatgcatcgtcaacattcttctgg----------------

D. miranda cgaaattaatttgacagccaacgcaaaacgcatgcatcgtcaacattcttctgg----------------

D. willistoni cgaaattaatttgacaaccaacgcaaaacgcatgcgtcgtcaacatttttcagc----------------

D. virilis cgaaattaatttgacaaccaacgcaaaacgcatgcgccttcaacattccacaac----------------

D. mojavensis cgaaattaatttgacaaccaacgcaaaacgcatgcgccgtcaacattccatgac----------------

D. melanogaster cgtggcggttgattacatgtacaaacgaaccgag

D. simulans cgtggcggttgattacatgtacaaacgaaccgag

D. sechellia cgtggcggttgattacatgtacaaacgaaccgag

D. yakuba cgtggcggttgattacacgtataaacgaagcgag

D. erecta cgtggcggttgattacacgtataaacgaacccag

D. biarmipes cggggcggttgattacatgaacaaacgaaccgaa

D. bipectinata cggggcggttgattacatgaacaaacgaaccgat

D. eugracilis cggggcggttgattacatgaacaaacgaaccgaa

D. elegans cggggcggttgattacatgaacaaacgaaccgaa

D. kikkawai cggggcggttgattacatgaacaaacgaaccgat

D. takahashii cggggcggttgattacatgaacaaacgaaccgaa

D. rhopaloa cggggcggttgattacatgaacaaacgaaccgaa

D. ficusphila cg-ggcggttgattacatgaacaaacgaaccgag

D. pseudoobscura cggggcggttgattacatgaacaaacgaaccgaa

D. persimilis cggggcggttgattacatgaacaaacgaaccgaa

D. miranda cggggcggttgattacatgaacaaacgaaccgaa

D. willistoni ctgggcggttgattaca-----------------

D. virilis ctcgacggttgattacatgaacaaacgaagcgaa

D. mojavensis ctggacggttgattacatgaacaaacgaagcgaa

D. melanogaster ggc--------ctacgtattaccggtggcaaatagtaattattacattaaatgcaccgcaa

D. simulans ggc--------ctacgtattaccggtggcaaatagtaattattacattaaatgcaccgcaa

D. sechellia ggc--------ctacgtattaccggtggcaaatagtaattattacattaaatgcaccgcaa

D. yakuba ggc--------ctacgtattaccggtggcaaatagtaattattacattaaatgccccgcaa

D. erecta ggc--------ctacgtattaccggtggcaaatagtaattattacattaaatgcaccgcaa

D. biarmipes ggc--------ctacgtattaccggtggcaaatagtaattattacattaaatgcaccgcaa

D. bipectinata ggc--------ctacgtattaccggtggcaaatagtaattattacattaaatgcaccgcaa

D. eugracilis ggc--------ctacgtattaccggtggcaaatagtaattattacattaaatgcaccgcaa

D. elegans ggc--------ctacgtattaccggtggcaaatagtaattattacattaaatgcaccgcaa

D. kikkawai ggc--------ctacgtattaccggtggcaaatagtaattattacattaaatgcaccgcaa

D. takahashii ggc--------ctacgtattaccggtggcaaatagtaattattacattaaatgcaccgcaa

D. rhopaloa ggc--------ctacgtattaccggtggcaaatagtaattattacattaaatgcaccgcaa

D. ficusphila ggc--------ctacgtattaccggtggcaaatagtaattattacattaaatgcaccgcaa

D. pseudoobscura ggc--------ctacgtattaccggtggcaaatagtaattattacattaaatgcaccgcaa

D. persimilis ggc--------ctacgtattaccggtggcaaatagtaattattacattaaatgcaccgcaa

D. miranda ggc--------ctacgtattaccggtggcaaatagtaattattacattaaatgcaccgcaa

D. willistoni ggctggttcctctacgtattaccagtggcaaatagtaattattacattaaatacacccaaa

D. virilis gcc--------acacgtattaccggtggcaaatagtaattattacattaaatgcaccgcaa

D. mojavensis gcc--------acacgtattaccggtggcaaatagtaattattacattaaatgcaccgcaa

D. melanogaster ccaaacttcaactc---cagccataaaagcaa

D. simulans ccaaacttcaactc---cagccataaaagcaa

D. sechellia ccaaacttcaactc---cagccataaaagcaa

D. yakuba ctaaacttcaactc---cagccataaaagcaa

D. erecta ccaaacttccactc---cagccataaaagcaa

D. biarmipes ccgaacttcaactc---cagccatgaaagcaa

D. bipectinata ctcaacttcaactc---cggccataaaaacaa

D. eugracilis ccaaacttcaactc---cagccataaaagcaa

D. elegans ccaaactccaactc---cagccataaaggcaa

D. kikkawai caaaacttcaactc---cggccataaaaacaa

D. takahashii ccaaacttcaactc---cagccataaaagcaa

D. rhopaloa ccaaacataaactc---cagccataaaagcaa

D. ficusphila ccaaacttcaactc---cagccataaaagcag

D. pseudoobscura ccaaacttcaactc---cagccataaaaacag

D. persimilis ccaaacttcaactc---cagccataaaaacag

D. miranda ccaaacttcaactc---cagccataaaaacag

D. virilis caggcagctcgcttcagcagcaacaacaacaa

D. mojavensis caggcagctcactt---cggccacagcaacat

D. melanogaster gaaacggcaaatgaagctctcaaagcgaactgtgctt--cgctggtggtccatt

D. simulans gaaacggcaaatgaagctctcaaagcgaactgtgctt--cgctggtggtccatt

D. sechellia gaaacggcaaatgaagctctcaaagcgaactgtgctt--cgctggtggtccatt

D. yakuba gaaacggcaaatgaagctctcaaagcgaactgtgctt--cgttggtggtccatt

D. erecta gaaacggcaaatgaagctctcaaagcgaactgtgctt--cgttggtggtccatt

D. biarmipes gaaacgacaaatgaagctctcaaagcgaactgcgctt--cgttggtggtccatt

D. bipectinata gaaacggcaaatgaagctctcaaagcaaactccactt--cgttggtggtccagt

D. eugracilis gaaacggcaaatgaagctctcaaagcgaactgtgctt--cgttggtggtccatt

D. elegans gaaacggcaaatgaagctctcaaagcaaactgcgctt--cgttggtggtccatt

D. kikkawai gaaacggcaaatgaagctctcaaagcaaactccgctt--cgttggtggtccagt

D. takahashii gaaacggcaaatgaagctctcaaagcgaactgagctt--cgttggtggtccatt

D. rhopaloa gaaacggcaaatgaagctctcaaagcaaactgcgctt--cgttggtggtccatt

D. ficusphila gaaacggcaaatgaagctctcaaagcaaactgcgctt--cgttggtggtccatt

D. pseudoobscura gaaacggcaaatgaagctctcaaagcaaactcctctcaacgttggtggtccagt

D. persimilis gaaacggcaaatgaagctctcaaagcaaactcctctcaacgttggtggtccagt

D. miranda gaaacggcaaatgaagctctcaaagcaaactcctctcaacgttggtggtccagt

D. willistoni gaaacggcaaatgaagctctcaa----------------cgttggtggtccagt

D. virilis gaaacggcaaatgaagctctcaa--------ccgcca--cgttggtggtccaat

D. mojavensis gaaacggcaaatgaagctctcaa-----ccgccgcca--cgttggtggtccaat

D. melanogaster ctgggga--------------gagcg------------------aaataaagctaaaatatgc

D. simulans ctgggga--------------gagcg------------------aaataaagctaaaatatgc

D. sechellia ctgggga--------------gagcg------------------aaataaagctaaaatatgc

D. yakuba cttggga--------------gagcg------------------aaataaagctaaaatatgc

D. erecta cttggga--------------gagcg------------------aaataaagctaaaatatgc

D. biarmipes cttgggagatcgg--------gagag------------------gaataaagctgaaatatgc

D. bipectinata ctgggga--------------gagcggctgatcgggagagcagtggataaagctaaaatatgc

D. eugracilis cttgggagatcggg------agagcg------------------taataaagctaaaatatgc

D. elegans cttgggagatcggg------agagcg------------------aaataaagctaaaatatgc

D. kikkawai catcggagagcgga------agagat------------------ggataaagctaaaatatgc

D. takahashii cttgggagatcgg--------gagag------------------gactaaagctaaaatatgc

D. rhopaloa cttgggagatcggg------agagcg------------------gaataaagctaaaatatgc

D. ficusphila ctcgggagatcggg------agagcg------------------gtataaagctaaaatatgc

D. pseudoobscura cttcagagagtaga--agagagagag--agagagagagagcaatggataaagctaaaatatgc

D. persimilis cttcagagagtagaagagagagagag--agagagagagagcaatggataaagctaaaatatgc

D. miranda cttcagagagtaga------agagag--agagagagagagcaatggataaagctaaaatatgc

D. melanogaster atgttggag----------------------------------aaaaaatgccgcccatgtcgccaaaat

D. simulans atgttggag---------------------------------aaaaaaatgccgcccatgtcgccaaaat

D. sechellia atgttggag---------------------------------aaaaaaataccgcccatgtcgccaaaat

D. yakuba atggtggag---------------------------------aaaaaaatgccgcccatgtcgccaaaaa

D. erecta atgttggag---------------------------------aaaaaaatgccgcccatgtcgccaaaat

D. biarmipes atgttggag----------------------------------aaaaaatgccgcccatgtcgccaaaat

D. bipectinata acgttggagctgctggggcaacaacaacaacaacaacaacagaaaaaaaggccgcccatgtcgccaaaat

D. eugracilis atgttggag---------------------------------aaaaaaatgccgcccatgtcgccaaaat

D. elegans atgttggag------------------------------aagaaaaaaatgccgcccatgtcgccaaaat

D. kikkawai aagttggag----------------------------------aaaaaatgccgcccatgtcgccaaaaa

D. takahashii atgttggaa----------------------------------aaaaaatgccgcccatgtcgccaaaat

D. rhopaloa atgttggag-------------------------------aagaaaaaatgccgcccatgtcgccaaaat

D. ficusphila atgttggag---------------------------------aaaaaaatgccgcccatgtcgccaaaat

D. pseudoobscura aagctgaag-----------------------ctgctatggcaaaaaaatgccgcccatgtcgccaaaaa

D. persimilis aagctgaag-----------------------ctgctatggcaaaaaaatgccgcccatgtcgccaaaaa

D. miranda aagctgaag-----------------------ctgctatggcaaaaaaatgccgcccatgtcgctaaaaa

D. melanogaster tttagcatc---ggaacatgcaaaaacagacatca-tcgcatggg--------------------gcagc

D. simulans tttagcatc---ggaacatgcaaaaacagacatca-tcgcatggg--------------------gcagc

D. sechellia tttagcatc---ggaacatgcaaaaacagacatca-tcgcatggg--------------------gcagc

D. yakuba cttagcatc---ggaacatgcaaaaacagacatca-gcgcatggg--------------------gcagc

D. erecta cttagcatc---ggaacatgcaaaaacagacatca-gcgcatggg--------------------ccagc

D. biarmipes tttagcatcg--ggaacatgcaaaaacagacatca-tcgcatggg--------------------gcagc

D. bipectinata tttagcatcg--ggaacatgcaaaaacagacatca-gcacatggggcaaaaacaataacaggaaagcaac

D. eugracilis tttagcatcg--ggaacatgcaaaaacagacatca-tcgcatgag------------------caacaac

D. elegans tttagcatcg--ggaacatgcaaaaacagacatca-gcgcatggg--------------------gcagc

D. kikkawai tttagcatcg--agaacatgcaaaaacagacatca-------aag--------------------gcagc

D. takahashii tttagcatcg--ggaacatgcaaaaacagacatca-tcgcatggg--------------------gcagc

D. rhopaloa tttagtatcg--ggaacatgcaaaaacagacatca-gcgcatgga--------------------gcagc

D. ficusphila tttagcatcg--ggaacatgcaaaaacaaacatca-gcgcattgg--------------------gctac

D. pseudoobscura ttttgtattgaaataacatgcaaaaacagacatca-gc----gag--------------------gcag-

D. persimilis ttttgtattgaaataacatgcaaaaacagacatca-gc----gag--------------------gcag-

D. miranda ttttgtattgaaataacatgcaaaaacagacatca-gc----gag--------------------gcag-

D. willistoni tttagcatta--aaaacatgcaa-----------------------------------------------

D. virilis tttagtatta--aaaacatgcaaaaacagacatcaagcccatgtg--------------------gcagc

D. mojavensis tttagtatta--aaaacatgcaaaaacagatatcagacgcatgtg--------------------gcagg

D. melanogaster --------------ta------------------------ggg-----------gaccacccacatcgct

D. simulans --------------tg------------------------ggg-----------gaccacccacatcgct

D. sechellia --------------tg------------------------ggg-----------gaccacccacatcgct

D. yakuba --------------t-------------------------ggg-----------gaccacccacaacgct

D. erecta --------------tg------------------------ggg-----------gaccacccacaacgct

D. biarmipes --------------tg------------------------ggg----------cgaccacccacaacgct

D. bipectinata --------------tggagagggagggaggtagaaggaacgga-----------gaccacccacaacgct

D. eugracilis gtgggggtacggtatg------------------------gcg-----------gaccacccactacgct

D. elegans --------------aggagagagccggcgagggt------tgg-----------gaccacccacaacgct

D. kikkawai --------------ta------------------------gtg-----------gaccacccacaacgct

D. takahashii --------------tg------------------------gggtggcgtggcgcgaccacccacaacgct

D. rhopaloa --------------aggagaggggcgaggggag-------gag-----------gaccacccacaacgct

D. ficusphila --------------tg------------------------ggg----------cgaccacccacaacgct

D. pseudoobscura --------------ct------------------------aag------gctacgaccacccacaacgcc

D. persimilis --------------ct------------------------aag------gctgcgaccacccacaacgcc

D. miranda --------------ct------------------------aag------gctacgaccacccacaacgcc

D. willistoni --------------tacgaaaccc----------------gag-----------gaccacccacaatgcc

D. virilis --------------ct------------------------ggg-----------gaccacccacaatgcc

D. mojavensis --------------cc------------------------ggg-----------gaccacccacaatgcc

D. melanogaster acatcgcttggcggtttcagtttaatgaaggcaga--

D. simulans acatcgcttggcggtttcagtttaatgaaggcaga--

D. sechellia acatcgcttggcggtttcagtttaatgaaggcaga--

D. yakuba acaacgcttggcggtttcagtttaatgaaggcagc--

D. erecta acaacgcttggcggtttcagtttaatgaaggcagc--

D. biarmipes acaacgctcggcggtttcggtttaatgaaggcagc--

D. bipectinata acaacgcttggcggtttcagtttaatgaaggcagc--

D. eugracilis actacgcttcgcgttttcagtttaatgaaggcagc--

D. elegans acaacgctcgacggtttcagtttgatgaaggcagc--

D. kikkawai acaacgcttggcggtttcagtttaatggaagcagc--

D. takahashii acaacgctcggcggtttcagtttaatgaaggcagc--

D. rhopaloa acaacgctcgacggtttcagtttaatgaaggcagc--

D. ficusphila acaacgctcggcggtttcagtttaatgaaggcagc--

D. pseudoobscura acaacgccggtcg--ttgagtttaatgaaggcaacaa

D. persimilis acaacgccggtcg--ttgagtttaatgaaggcagcaa

D. miranda acaacgccggtcg--ttgagtttaatgaaggcaac--

D. willistoni acaatgccggccg--ttgagtttaatgaaggcaac--

D. virilis acaatgccggcag--ccgcttttaatgaa-gcaac--

D. mojavensis acaatgccggcag--ccgcttttaatgaa-gcaac--

D. melanogaster --------tttttgggtggtccacac----tgcagcg--aaaa-------taa----actacagtggcaa

D. simulans --------tttttgggtggtccacac----tgcaacg--aaaa-------taa----accacagtggcaa

D. sechellia --------tttttgggtggtccacac----tgcaacg--aaaa-------taa----accacagtagcaa

D. yakuba --------tttttgggtggtccacac----tgcaacg--aaaa-------taa----accacagtggcaa

D. erecta --------tttttgggtggtccccac----tgcagcg--aaaa-------taa----accacagtggcaa

D. biarmipes ccgaaagcactttgggtggtctttgc----gaaaata--aaaa-------taatggcacgcgaataacaa

D. bipectinata --------tttttgggtggtccccgg----tgccatg--aaaa-------taa----accgccgcag--a

D. eugracilis --------tttttgggtggtccacac----tgcaatg--aaaa-------taa----accaccgtggaaa

D. elegans --------attttgggtggtccacac----tgcaatg--gaaa-------taa----accaccgtggcaa

D. kikkawai --------tttttgggtggtccacac----tgcagtgaaaaaa-------taa----agcaccgcagcaa

D. takahashii --------tttttgggtggtccacac----tgcaata--aaaa-------taa----accaccgtggcaa

D. rhopaloa --------tttttgggtggtccacac----tgcaatg--aaaaaagaaagaaa----accaccgtggcaa

D. ficusphila --------tttttgggtggtccatccacactgcaat---aaaa-------taa----accaccgtggcag

D. pseudoobscura --------tttttgggtggtc--tgc----tacaaca--acaa-------tca-----------------

D. persimilis ---------ttttgggtggtc--tgc----tacaaca--acaa-------tca-----------------

D. miranda --------tttttgggtggtc--tgc----tacaaca--acaa-------tca-----------------

D. willistoni --------tatttgggtggtcc-----------------acca-------cca-----------------

D. virilis --------tatttgggtggtc---gc----tgcaata--aaaa-------taa--------caacaacat

D. mojavensis --------tatttgggtggtc---gc----tgcaata--aaaa-------taa--------cagcaacaa

D. melanogaster caac---------aaaccagc-agc----ca-aggcacttt---gggtggtccatgcaaaa---------

D. simulans caac---------aaaccagc-agc----cg-aagcacttt---gggtggtccgtgcaaaa---------

D. sechellia caac---------aaactagc-agc----ca-aagcacttt---gggtggtccgtgcaaaa---------

D. yakuba caac---------aaaccagc-agc----ca-aagcacttt---gggtggtccgtgcaaaa---------

D. erecta caac---------aaaccagc-agc----ca-aagcacttt---gggtgggccgtgcaaaa---------

D. biarmipes caat-------agaaattagcgagctccgca-gtgaatgttggaggggagttagctcgata---------

D. bipectinata aaag---------gaaacaga-aac-------ggccgtttt---gggtggtctttgaaaat---------

D. eugracilis caacaactacaaaaaaacagc-agc----cagaagcacttt---gggtggtct-tgcgaat---------

D. elegans catcaacaaca--aaaacagc-agc----caaaagcacttt---gggtggtctcggcaaaa---------

D. kikkawai caacaatagca--aaagcggc-agc-----a-aaatacttc---g----------ccaaaaaccaaaatc

D. takahashii caac----acaaaaaagcagc-agc----cgaaagcacttt---gggtggtctttgcgaaa---------

D. rhopaloa catcaacaaca--aaaacagc-agc----caaaagcacttt---gggtggtctcgccaaaa---------

D. ficusphila caaaaaaa-----aaaacagc-agc----ca-aatctcttt---aggtggtctcggcgaaa---------

D. pseudoobscura -------------aaa------agc----ct-------ttt---gggtggtcgtggcaaca---------

D. persimilis -------------aaa------agc----ct-------ttt---gggtggtcgtggcaaca---------

D. miranda -------------aaa------agc----ct-------ttt---gggtggtcgtggcaaca---------

D. willistoni -------------------gc-aac----ct-----cgctt---gggtggtc----caaac---------

D. virilis gaata--------acgccagc-ggg----cg-cag--tcgt---gtgtggtcttagctaaa---------

D. mojavensis caacaacaggaatacgcctgc-ggg----cg-caat-tcgt---gtgtggtctaagctaca---------

**Enhancer 1EH**

D. melanogaster gaaccc--aatggcccaca------taactcc

D. simulans gaaccc--aatggcccaca------taactcc

D. sechellia gaaccc--aatggcccaca------taactcc

D. yakuba gagccc--aatggcccaca------taactcc

D. erecta gaaccc--aatggcccaca------taactcc

D. biarmipes gagccc--aatggcccaca------taactcc

D. bipectinata gaaccc--aatggcccacatacacgtaacacc

D. eugracilis gaaacc--aatggcccaca------taactcc

D. elegans gacacc--aatggcccaca------taactcc

D. kikkawai gagccccgaatggcccacatata--taactcc

D. takahashii gaaccc--aatggcccaca------taactcc

D. rhopaloa gatccc--agtggcccaca------taactcc

D. ficusphila gagccc--aatggcccaca------taactcc

D. pseudoobscura ataccc--aatggcccaca------taacacc

D. miranda ataccc--aatggcccaca------taacacc

D. willistoni taaacc--tttgccccaca------taacacc

D. virilis ggagtc--agtgccccaaa------tagcacc

D. melanogaster acaac--------------------------gg------------cccgac------------------a

D. simulans acaac--------------------------gg------------cccgac-------------------

D. sechellia acaac--------------------------gg------------cccgac-------------------

D. yakuba acaac--------------------------gg------------ctcgac-------------------

D. erecta acaac--------------------------ag------------cccgac-------------------

D. biarmipes acaac--------------------------gg------------c-cgac-------------------

D. bipectinata acgac--------------------------ga--------agaccccgaa-------------------

D. eugracilis acaac--------------------------gg------------gccgacacatagggagagagagaga

D. elegans acaac--------------------------gg------------cccaag-------------------

D. kikkawai -------------------------------ga---------------gac-------------------

D. takahashii acaac--------------------------gg------------cccgac-------------------

D. rhopaloa acaac--------------------------gg------------cccgag-------------------

D. ficusphila acaac--------------------------gg------------cccgaa-------------------

D. pseudoobscura acagc--------------------------aa--------------caac-------------------

D. miranda acagc--------------------------aa--------------caac-------------------

D. willistoni acagc--------------------------agaaagaaagagagacccaa-------------------

D. melanogaster gagagagagag---------------------agaa-----------------gaa

D. simulans ---agagagag---------------------agaa-----------------gaa

D. sechellia ---agagagag---------------------agaa-----------------gaa

D. yakuba ---agcgagag---------------------agaa-----------------gaa

D. erecta ---a--gagag---------------------agaa-----------------gaa

D. biarmipes ---agagagag---------------------agaa-----------------g-a

D. bipectinata --gagaaagag---------------------agaa-----------------gaa

D. eugracilis gagagagagagacagagagagagggagagagaagaa-----------------gaa

D. elegans ---agagagagaca------------------agaa-----------------gaa

D. kikkawai ---a-----ag---------------------agga-----------------gaa

D. takahashii ---agagagag---------------------agaa-----------------gaa

D. rhopaloa ---agagagag---------------------agaa-----------------g-a

D. ficusphila gaaagagagag---------------------agaa-----------------gaa

D. pseudoobscura ---a-aaagag---------------------agaa-----------------gaa

D. miranda ---a-aaagag---------------------agaa-----------------gaa

D. willistoni ---agaaaaaa---------------------agaa-----------------aaa

D. melanogaster cagtgacagagag------cgaatacc

D. simulans cagtgacagagag------cgaatacc

D. sechellia cagtgacagagag------cgaatacc

D. yakuba cagtgacagagag------cgaatacc

D. erecta cagtgacagagag------cgaatacc

D. biarmipes cagtgacagagag------cgaatacc

D. eugracilis cagtgacagagag------cgaatacc

D. elegans cagtgacagagag------cgaatacc

D. kikkawai cagtgacagagag------cgaatacc

D. takahashii cagtgacagagag------cgaatacc

D. rhopaloa cagtgacagagag------cgaatacc

D. ficusphila cggtgacagagag------cgaatacc

D. pseudoobscura gagtggaagagagggggcccgaatacc

D. miranda gagtggaagagagggggcccgaatacc

D. melanogaster ctagtcacgttttcggggcgccataaatt

D. simulans ctagtcacgttttgggggcgccataaatt

D. sechellia ctagtcacgttttgggggcgccataaatt

D. yakuba ctagtcacgttttgggggcgccatatatt

D. erecta ctagtcacgttttgggggcgccataaatt

D. biarmipes ctagtcacgtttc-ggggcgccataaatt

D. bipectinata ctagtcacgttttgggggcgccataaatt

D. eugracilis ctagtcacgttttgggggcgccataaatt

D. elegans ctagtcacgttttgggggcgccataaatt

D. kikkawai ctagtcacgttttgggggcgccataaatt

D. takahashii ctagtcacgttttgggggcgccataaatt

D. rhopaloa ctagtcacgtttcgggggcgccgtaaatt

D. ficusphila ctagtcacgttttgggggcgccataaatt

D. pseudoobscura ctagtcacgttttgggggcgccataaatc

D. miranda ctagtcacgttttgggggcgccataaatc

D. willistoni ctagtcacgttttgggggcgccataaatt

D. virilis ctggtcacgttttaggggcgccataaatt

D. mojavensis ccagtcacgtttaaggggcgccataaatt

D. melanogaster ct--acaagttttatggcacacg-c

D. simulans ct--acaagttttatggcacacg-c

D. sechellia ct--acaagttttatggcacacg-c

D. yakuba ct--acaagttttatggcacacg-c

D. erecta ct--acaagttttatggcacacg-c

D. biarmipes ct--acaagttttatggcacacg-c

D. bipectinata ct--acaagttttatggcacacg-c

D. eugracilis ct--acaagttttatggcacacg-c

D. elegans ct--acaagttttatggcacacg-t

D. kikkawai ct--acaagttttatggcacacg-t

D. takahashii ct--acaagttttatggcacacg-c

D. rhopaloa ct--acaagttttatggcacacg-c

D. ficusphila ct--acaagttttatggcacacg-c

D. pseudoobscura ct--acaagttttatggcacacg-t

D. miranda ct--acaagttttatggcacacg-t

D. willistoni ctacacaggttttatggcacacgtc

D. virilis ct--acacgtttaatggcacaca-t

D. mojavensis ct--acacgttttatggcacact-t

D. melanogaster tcgtctgggtggtctgccatataga----------

D. simulans tcgtctgggtggtctgccatattga----------

D. sechellia tcgtctgggtggtctgccatattga----------

D. yakuba tcgtctgggtggtctgccatattga----------

D. erecta tcgtctgggtggtctgccatattga----------

D. biarmipes gtgtctgggtggtctgccatattga----------

D. bipectinata gcctctgggtggtctgccatattga----------

D. eugracilis atatctgggtggtctgccatattga----------

D. elegans ggatct-ggtggtctgtcatattga----------

D. takahashii gtgtctgggtggtctgccatattga----------

D. rhopaloa ggatctgggtggtctgccatattga----------

D. ficusphila ---tctgggtggtctgccatattga----------

D. pseudoobscura gcctttgggtggtctgccatattga----------

D. miranda gcctttgggtggtctgccatattga----------

D. virilis tcggctgggtggtccgtcacgtcgc----------

D. mojavensis ttcactgggtggtccggcacgttgctgcttattga

D. melanogaster cggcataactgggtgcgctcatatt------taatggcc

D. simulans cggcataactgggtgcgctcatatt------ttatggcc

D. sechellia cggcataactgggtgcgctcatatt------ttatggcc

D. yakuba cggcataactgggtgcgctcatatt------ttatggcc

D. erecta cggcacaactgggtgcgctcatatt------ttatggcc

D. biarmipes cggcacaactgggtgcgctcatact------ttatggcc

D. bipectinata cggcataactgggtgcgctcatatt------ttatggcc

D. eugracilis cggcataactgggtgcgctcatatt------ttatggcc

D. elegans cggcataactgggtgcgctcgtatt------ttatggcc

D. kikkawai cggcataactgggtgcgctcatattttatggttatggcc

D. takahashii cggcataactgggtgcgctcatatt------ttatggcc

D. rhopaloa cggcataactgggtgcgctcatgtt------ttatggcc

D. ficusphila cggcataactgggtgcgctcatatt------ttatggac

D. pseudoobscura cggcataactgggtgcgctcatatt------ttatggct

D. miranda cggcataactgggtgcgctcatatt------ttatggct

D. melanogaster agc-ggt--------------ccgtgtaattaagtagtcagg-cgctgtggggggcc

D. simulans agc-ggt--------------ccgtgtaattaagtagtcagt-cgctgtggggggcc

D. sechellia agc-ggt--------------ccgtgtaattaagtagtcagg-cgctgtgtggggcc

D. yakuba agc-ggt--------------ccgtgtaattaagtagtcagg-cgctgtggggggcc

D. erecta agc-ggt--------------ccgtgtaattaagtagtcggg-cgctctggggggcc

D. biarmipes agc-ggg--------------ccgagtaattaagtagtcaag-cgctgtggggggcc

D. bipectinata atc-ggg--------------tcgtgtaattaagtagtcatt-ctctgtggggggtt

D. eugracilis agc-ggt--------------ccgtgtaattaagtagtcaag-cgctgtggggggcc

D. elegans agc-ggc--------------ccgtgtaattaagtagtcaag-cgctgtggggggcc

D. kikkawai agc-gga--------------ccgtgtaattaagtagtcagatctctgtggggggta

D. takahashii agc-ggg--------------ccgtgtaattaagtagtcaag-cgctgtgggggggc

D. rhopaloa ----ggc--------------ccgtgtaattaagtagtcaag-cgctgtggggggcc

D. ficusphila agc-ggt--------------ccgtgtaattaagtagtcaac-caccgtggggggcc

D. pseudoobscura atcgggg--------------ccgtgtaattaagtagtcaag-ctctgtgggggggc

D. miranda atcgggg--------------ccgtgtaattaagtagtcaag-ctctgtgggggggc

D. virilis ---------------------ccgtgtaattaagtagtcaaa-gcccgtggggggtg

D. mojavensis gtc-gactatttataatgcttcggtgtaattaagtagtcaaa-gcccgtgaggcatt

D. melanogaster gtcgga-tcttttgattgattgatgcgtg

D. simulans gtcgga-tcttttgattgattgatgcgcg

D. sechellia gtcgga-tcttttgattgattgatgcgtg

D. yakuba gtc-gg-tcttttgattgattgatgcgtg

D. erecta gtcggg-tcttttgattgattgatgcgtg

D. biarmipes gtcgga-tcttttgattgattgatgcgtg

D. bipectinata gtcgga-ta-tatgattgattgatgggga

D. eugracilis gtcgga-tcttttgattgattgatgcgtg

D. elegans gtcgga-ccttttgattgattgatgcgtg

D. kikkawai gtcgga-tc-tttgattgattgatgcgtg

D. takahashii gtcgga-tcttttgattgattgatgcgtg

D. rhopaloa gtcgga-tcttttgattgattgatgcgtg

D. ficusphila gtcggg-tcttttgattgattgatgcgtg

D. pseudoobscura gtcggt-tcttttgattgattgacggctg

D. miranda gtcggt-tcttttgattgattgacggctg

D. virilis cacgaa-ctctttgattgattagtgcgtt

D. mojavensis cacgaacctttttgattgattagagtgtt
